# Reprogramming of RNA silencing triggered by *cucumber mosaic virus* infection in Arabidopsis

**DOI:** 10.1186/s13059-021-02564-z

**Published:** 2021-12-15

**Authors:** Maria Luz Annacondia, German Martinez

**Affiliations:** Department of Plant Biology, Uppsala BioCenter, Swedish University of Agricultural Sciences and Linnean Center for Plant Biology, Uppsala, Sweden

**Keywords:** RNA silencing, Virus, Argonaute, Immunoprecipitation, Cucumber mosaic virus

## Abstract

**Background:**

RNA silencing has an important role mediating sequence-specific virus resistance in plants. The complex interaction of viruses with RNA silencing involves the loading of viral small interfering RNAs (vsiRNAs) into its host ARGONAUTE (AGO) proteins. As a side effect of their antiviral activity, vsiRNAs loading into AGO proteins can also mediate the silencing of endogenous genes. Here, we analyze at the genome-wide level both aspects of the interference of cucumber mosaic virus (CMV) with the RNA silencing machinery of Arabidopsis thaliana.

**Results:**

We observe CMV-derived vsiRNAs affect the levels of endogenous sRNA classes. Furthermore, we analyze the incorporation of vsiRNAs into AGO proteins with a described antiviral role and the viral suppressor of RNA silencing (VSR) 2b, by combining protein immunoprecipitation with sRNA high-throughput sequencing. Interestingly, vsiRNAs represent a substantial percentage of AGO-loaded sRNAs and displace other endogenous sRNAs. As a countermeasure, the VSR 2b loaded vsiRNAs and mRNA-derived siRNAs, which affect the expression of the genes they derive from. Additionally, we analyze how vsiRNAs incorporate into the endogenous RNA silencing pathways by exploring their target mRNAs using parallel analysis of RNA end (PARE) sequencing, which allow us to identify vsiRNA-targeted genes genome-wide.

**Conclusions:**

This work exemplifies the complex relationship of RNA viruses with the endogenous RNA silencing machinery and the multiple aspects of virus resistance and virulence that this interaction induces.

**Supplementary Information:**

The online version contains supplementary material available at 10.1186/s13059-021-02564-z.

## Background

The development and survival of plants depend on their ability to adapt to the surrounding environmental conditions, which can be beneficial or harmful. Changes in the surrounding environment of plants can occur suddenly; hence, plants rely on the plasticity of their genome to cope with these disturbances [1–3]. One of the genetic tools that helps plants to cope with stress is an adaptive and highly specific mechanism to regulate gene expression, termed RNA interference (RNAi) or RNA silencing [4, 5]. Briefly, this mechanism is triggered by the presence of a double-stranded RNA (dsRNA), which is cleaved by Dicer-like (DCL) proteins to produce small interfering RNAs (siRNAs), typically of 21- to 24-nt long. One strand of these siRNAs is then loaded into ARGONAUTE (AGO) proteins forming the RNA-induced silencing complex (RISC) to lead the posttranscriptional gene silencing (PTGS) or transcriptional gene silencing (TGS) of the target RNA [6]. siRNAs can initiate the amplification of the RNA silencing signal inducing the biogenesis of additional siRNAs termed secondary [7].

Viruses are an especially interesting group of pathogens as they entirely depend on their hosts to complete their life cycle [8]. Plants use the RNA silencing machinery as an antiviral strategy that provides sequence specificity against viruses [9, 10]. Once the virus has entered into the cell, dsRNAs produced from different sources, such as the viral genome, in the case of RNA viruses, or the transcripts produced by the viral genome, in the case of DNA viruses, trigger the activation of the RNA silencing pathway [6]. Then, DCL proteins process these dsRNAs into viral-derived small interfering RNAs (vsiRNAs), which are mainly 21-nt long and produced by DCL4 [6, 11, 12] or 22-nt long and produced by DCL2 [12–14] and immunize cells against the viral infection. Further evidence of the important role of RNA silencing as an antiviral mechanism is that to counteract this defense mechanism, viruses have developed proteins termed viral suppressors of RNA silencing (VSRs) [15, 16]. VSRs from different viruses can suppress the host RNA silencing activity at different steps of the pathway [15–17]. A common strategy, followed by multiple viruses, is the VRS-mediated sequestering of vsiRNAs to avoid the targeting of the viral genome by AGO proteins [15, 16]. This strategy is used by two of the most studied VSRs: the *Tombusvirus* P19 and the *Cucumovirus* 2b proteins. P19 is capable of binding vsiRNAs to avoid the antiviral activity of the RNA silencing pathway [18, 19]. Furthermore, P19 can bind endogenous sRNAs like the microRNA (miRNA) miR168, which modulates AGO1 mRNA levels during viral infection [19]. 2b loads vsiRNAs to prevent the RNA silencing signal spreading and, ultimately, the silencing of the viral genome [20]. Additionally, 2b can inhibit PTGS and TGS by interfering with the function of AGOs by blocking AGO1 cleavage activity and, therefore, inhibit miRNA pathways [21], and by directly interacting with AGO4 and interfering with the RNA-directed DNA methylation pathway [20]. These proteins exemplify the duality of the interaction of viruses with the RNA silencing machinery, which is used by the host to control viral accumulation, but can also be used by the virus to regulate host processes.

In the last years, this dual interaction with the RNA silencing pathway has been studied in several host/parasite interactions. A common theme for many pathogens is the development of an alternative interaction with the host RNA silencing machinery that allows them to target host endogenous genes. Examples of this side-effect been described in different types of pathogens, such as parasitic plants, fungi, viruses, or viroids [22–25] and endogenous genomic parasites such as transposable elements [26]. For instance, the parasitic plant *Cuscuta campestris* produces 22-nt miRNAs that target *Arabidopsis thaliana* genes during their parasitic interaction, causing a decrease on the expression of host genes, such as auxin receptors or phloem proteins, among others [22]. Moreover, the fungal pathogen *Botrytis cinerea* produces sRNAs that target *Arabidopsis* genes related to plant immunity, such as *MITOGEN-ACTIVATED PROTEIN KINASE 1* and 2 (*MPK1* and *MPK2*); *PEROXIREDOXIN IIF* (*PRXIIF*), a gene involved in oxidative stress; and *CELL WALL-ASSOCIATED KINASE* (*WAK*), by binding to the host AGO1 [23]. In the case of viruses and viroids, they use the vsiRNAs (vdsiRNAS for viroids) generated by the RNA silencing pathway on their own benefit by targeting host genes [24, 25, 27, 28]. This is the case of CMV Y-satellite RNA (Y-Sat), a small parasitic RNA that accompanies CMV and produces vsiRNAs that can target and downregulate a *Nicotiana benthamiana* gene involved in chlorophyll biosynthesis (I subunit of Mg-chelatase, *CHLI*) [24, 27]. Notably, although selected examples of genes targeted by vsiRNAs have been described, the genome-wide effects of vsiRNAs on host gene expression have not been characterized yet.

To shed light into that, here, we analyzed the interference of CMV with the RNA silencing pathways of *Arabidopsis thaliana*. To this aim, we studied the viral and endogenous sRNA populations in CMV-infected *Arabidopsis* plants, their loading into the main antiviral AGOs (AGO1, AGO2, AGO5, and AGO7) and the CMV VSR protein 2b, and the vsiRNA-targeted genes by means of parallel analysis of RNA ends (PARE). Our analysis revealed that the loading of vsiRNAs into AGO proteins displaced host endogenous sRNAs but also that vsiRNAs targeted a myriad of endogenous genes, mainly photosynthesis-related. Furthermore, 2b loaded vsiRNAs together with host mRNA-derived sRNAs which might interfere with the transcriptional regulation of their mRNAs of origin. In summary, our study provides a complete analysis of the interaction of a viral infection with the host RNA silencing machinery and sheds light into the multiple aspects of RNA silencing affected by RNA pathogens.

## Results

### CMV infection disrupts the *Arabidopsis* endogenous sRNA populations

**Fig. 1:**
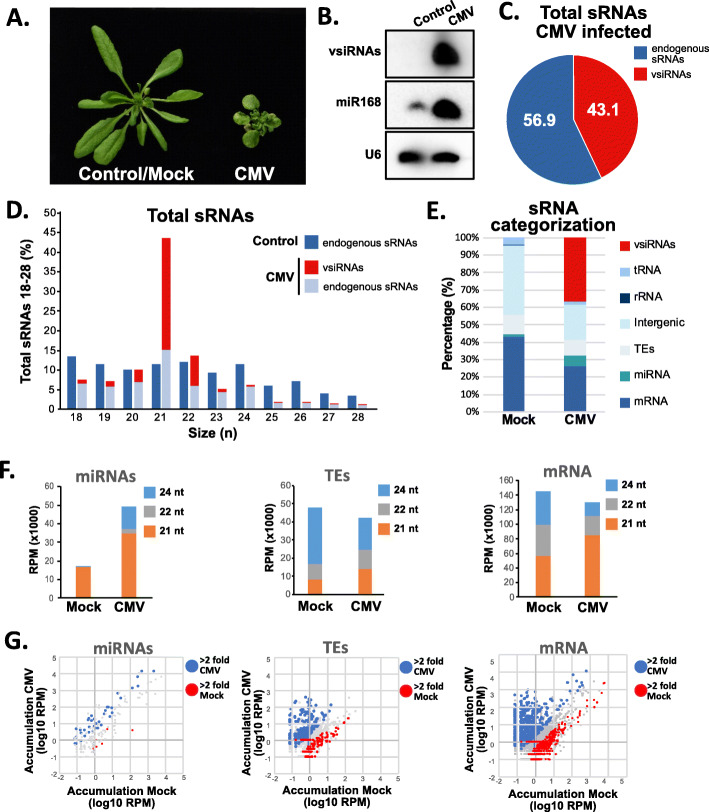
CMV-derived sRNAs affect endogenous sRNA proportions. **A** Representative pictures of mock and CMV-infected *Arabidopsis* Col-0 at 20 dpi. **B** Northern blot detection of vsiRNAs, miR168, and U6. U6 was used as a loading control. 20 μg of total RNA was loaded on each lane. **C** Proportion of CMV sRNAs vs endogenous *Arabidopsis* sRNAs in CMV-infected sRNA libraries. **D** Size distribution of the whole mapped sRNAs for mock and CMV-infected libraries. **E** Categorization in different classes of sRNA for mock and CMV-infected samples. **F** Proportion of 21-, 22-, and 24-nt sRNAs mapping to miRNAs, TEs, and mRNAs in mock and CMV-infected samples. **G** Scatterplot of sRNA accumulation (log10 of RPMs) for individual miRNA families, TEs, and mRNAs. Elements that accumulate 2-fold or higher sRNAs in CMV samples are highlighted in blue while elements that accumulate 2-fold or higher in mock samples are highlighted in red. Two bioreplicates from sRNA libraries were generated and analyzed

To understand how sRNA populations are affected during CMV infection in *Arabidopsis*, we produced and sequenced sRNA high-throughput libraries of both mock and CMV *Fny*-inoculated rosette leaves at 30 dpi (Additional file 1: Table S1 and Fig. 1A). As previously described, vsiRNAs accumulated to high levels in CMV-infected tissues [29], comparable to endogenous miRNAs with high accumulation levels like miR168 (Fig. 1B). The analysis of our sRNA data identified that in our libraries vsiRNAs represented almost half (43.1%) of the total pool of sRNAs that were sequenced, being the rest derived from the *Arabidopsis* genome (56.9%) (Fig. 1C). This considerable population of vsiRNAs was mainly constituted by vsiRNAs that were 21-nucleotide (nt) long (70.2%), followed by 22-nt (18.14%) and 20-nt vsiRNAs (7.5%), as it was previously reported for RNA viruses in different studies [29–31] (Fig. 1D). Moreover, vsiRNAs were produced from the three genomic RNAs that constitute the CMV genome, predominantly from RNA 2 (42.94%) and RNA 3 (41.94%), with a lower proportion coming from RNA 1 (15.11%) (Additional file 2: Fig. S1A-B). This proportion of vsiRNAs from the different genomic RNAs at our sampling time (30 dpi) was confirmed by Northern blot (Additional file 2: Fig. S1C). Interestingly, vsiRNAs followed a different dynamic of accumulation than genomic RNAs, which were relatively stable at 10, 20, and 30 dpi (Additional file 2: Fig. S1C). This difference of accumulation between genomic and siRNAs from CMV probably is due to the effect of RDR-mediated amplification of the RNA silencing signal [14, 32] as previously reported for CMV [33]. Next, to understand how vsiRNAs affected the different endogenous sRNA populations, we analyzed the proportion of different classes of sRNAs in both mock and CMV-infected sRNA libraries (Fig. 1E and Additional file 2: Fig. S2). In general, infection led to a general reduction in the accumulation of endogenous sRNAs, with the exception of miRNAs, which increased their accumulation (Fig. 1E). We further confirmed this higher accumulation levels for miR168 by Northern blot (increased more than 32-fold in CMV libraries, as previously described [21, 34], Fig. 1B). A detailed analysis of sRNA profiles showed that together with miRNAs, 21-nt sRNAs derived from mRNAs and TEs also increased their accumulation in infected tissues (Fig. 1F and Additional file 2: Fig. S2). 22.3% of miRNA families accumulated to higher level under CMV infection (>2 fold with *p* value<0.05), including important developmental regulators like miR158, miR172, or miR393 (Fig. 1G and Additional file 1: Table S2). On the other hand, 6.8% and 6.7% of mRNAs and TEs, respectively, produced increased amount of 21-nt sRNAs under CMV infection (>2fold with *p* value<0.05, Fig. 1G and Additional file 1: Table S3 and Additional file 1: Table S4). TEs producing 21-nt sRNAs during CMV infection were significantly enriched in members of the Gypsy superfamily and depleted in members of the DNA superfamily (Additional file 2: Fig. S3A). Gypsy members occupy heterochromatic regions in the *Arabidopsis* genome [35], and, in line with the enrichment of this class of TEs, TEs producing a higher amount of 21-nt sRNAs during CMV infection had heterochromatic features including significant higher values of DNA methylation, size, and of the heterochromatic histone modification H3K9me2 (Additional file 2: Fig. S3B-D). This higher accumulation of 21-nt sRNAs might be due to their transcriptional reactivation, since 24-nt TE-derived sRNAs were reduced during CMV infection (Additional file 2: Fig. S2D). Additionally, the production of 21-nt TE-derived sRNAs could be attributed to the activity of the VSR of CMV, 2b, since the increased production of TE-derived sRNAs during CMV infection was absent in data obtained from *Arabidopsis* plants infected with the CMV-∆2b strain [33, 36], which lacks the 2b protein (Additional file 2: Fig. S3E). Altogether, these results showed that vsiRNAs derived from CMV were produced from all its genome entirety, were mainly 21-nt long, and potentially affected the proportion of endogenous sRNA populations due to their relative high accumulation levels.

### Global AGO sRNA-loading is affected by vsiRNAs

**Fig. 2:**
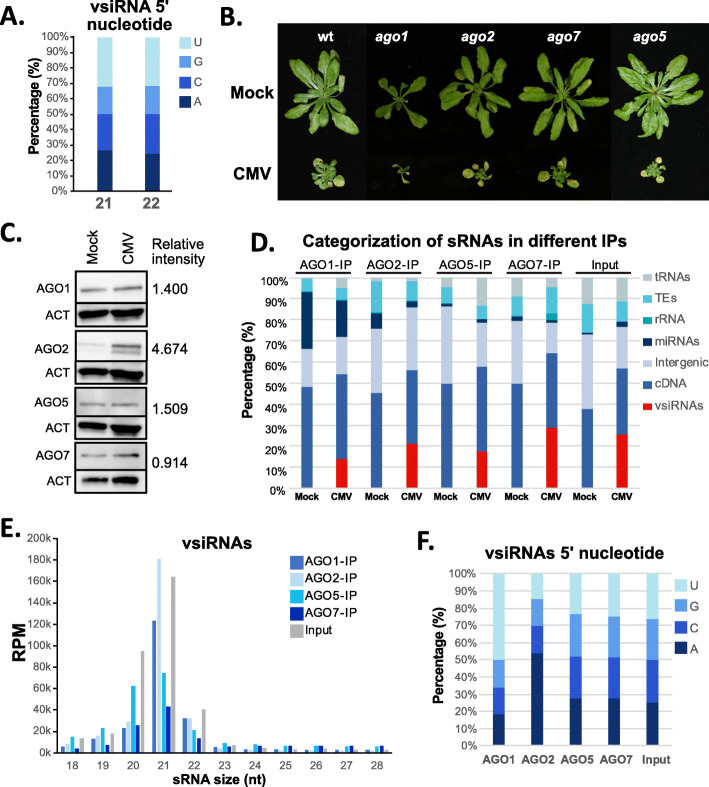
vsiRNA incorporation into AGO proteins. **A** Proportion of 5′ nucleotide distribution in 21- and 22-nt vsiRNAs. **B** Representative pictures of CMV-infected AGO mutants. **C** Western blot showing the accumulation of AGO proteins during CMV infection. Two biological replicates were used for the calculation of the relative intensity of the bands. **D** sRNA categorization for AGO-IPed sRNA libraries in mock and CMV-infected tissues. **E** Size distribution of the vsiRNAs loaded into the different studied AGOs. **F** Proportion of 5′ nucleotide distribution in 20- to 22-nt vsiRNAs for the different AGO-IPed sRNA libraries. Two bioreplicates from each AGO-IPed sRNA libraries were generated and analyzed

To better understand the potential hijacking of the host RNA silencing machinery caused by vsiRNAs, we studied the interaction of vsiRNAs with the host AGO proteins, the key effectors of the antiviral pathway. Since AGO proteins load sRNAs with a preference for certain 5′ nucleotides [37], we initially inferred the potential preference of vsiRNAs by analyzing their 5′ nucleotide prevalence in our sRNA sequencing data (Fig. 2A). 21 and 22-nt vsiRNAs had a similar 5′ nucleotide distribution with U being the main 5′ nucleotide (31.8 and 31.4%, respectively) followed by A (26.6 and 24.3%), C (23.7 and 25.8%), and G (17.8 and 18.3%), indicating a preference for a loading into AGO1, AGO2, and AGO5 [37]. In line with their previously described antiviral role, mutants in AGO1, AGO2, and AGO5 were significantly susceptible to CMV *Fny* infection compared to mock plants (Fig. 2B and Additional file 2: Fig S4A), while AGO7 mutants were slightly resistant to the infection (Additional file 2: Fig S4A). During CMV infection, all the AGO proteins analyzed, but AGO7, increased their accumulation, especially AGO2 (4.6 fold increase relative to mock (Fig. 2C and Additional file 2: Fig S4B) as previously described [38].

Next, we analyzed the characteristics and disturbance of sRNA loading into AGO proteins during CMV infection. To that end, we performed AGO immunoprecipitation (IP) followed by sRNA high-throughput sequencing for AGO1, AGO2, AGO5, and AGO7 (all of which have been showed to have an antiviral role) [4, 11, 38, 39], in mock- and CMV-inoculated plants using commercial antibodies for AGO1, AGO2, and AGO5 and an AGO7-HA tagged transgenic line (AGO7:HA-AGO7) transformed in an *ago7* mutant [40]. Our AGO-IP analysis showed interesting characteristics of AGO behavior during viral infection (Fig. 2D). First, CMV infection induced vsiRNA loading in all AGO analyzed (Fig. 2D). In terms of total AGO sRNA proportion, vsiRNAs occupied mainly AGO7 (28.9%), AGO2 (21.3%), AGO5 (17.7%), and AGO1 (13.9%) (Fig. 2D). vsiRNAs loaded into AGO proteins were mainly on 21-nt in AGO1 and AGO2 (56.3 and 64.2% of all 18- to 28-nt vsiRNAs, Fig. 2E), while AGO5 and AGO7 loaded both 20- and 21-nt vsiRNAs (26.1/31.2% and 19.4/32.4% of 20/21-nt sRNAs, Fig. 2E). Interestingly, the overall input vsiRNA profile (normalized to reads per million, RPM) presented prominent peaks of 20- and 21-nt length, resembling a compromise between both AGO1 and AGO2 21-nt and AGO5 and AGO7 20-/21-nt preferential vsiRNA loading (Fig. 2E). AGO2 and AGO1 were the AGO proteins with the higher vsiRNA loading, as expected, while AGO5 and AGO7 had a secondary role in the antiviral response (Fig. 2E), similar to the role of AGOs during *Turnip mosaic virus* infection (TuMV) in *N. benthamiana* [4]. vsiRNAs loaded into different AGOs presented the expected 5′ nucleotide preference previously described in *Arabidopsis* [37], with AGO1 loading 57.5% and AGO2 loading 60% of U and A 5′-nucleotide terminal 20–22-nt vsiRNAs, respectively (Fig. 2F). In parallel to vsiRNA loading, other sRNA categories decreased their AGO occupancy, specially miRNAs in AGO1, AGO2, and AGO7 (1.5-, 2.6-, and 2-fold decrease, respectively). Unexpectedly, AGO5 increased the loading of specific miRNA families under CMV infection (global 1.5-fold increase Fig. 2D and Additional file 2: Fig S5). Together with miRNAs, the general trend for the rest of genomic categories was a decrease of their accumulation. This was the case for mRNA-, TE-, and intergenic-derived sRNAs (1.3-, 1.2-, and 1.5-fold decrease on average for the four AGOs under study, Fig. 2D). In summary, CMV infection affected all AGO protein homeostasis, first increasing AGO protein accumulation (except AGO7) but also affecting the sRNA proportions loaded in AGO proteins, which might be displaced by vsiRNA loading.

### 2b VSR loads 20–21 nt viral and mRNA-derived sRNAs

**Fig. 3:**
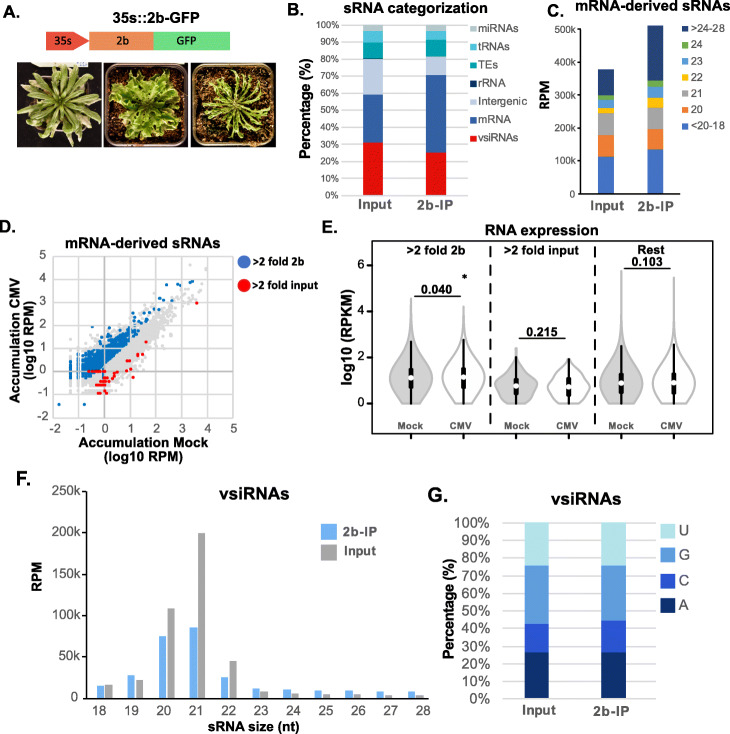
CMV 2b binds endogenous sRNAs. **A** Representative pictures of 2b-GFP overexpression transgenic lines. **B** sRNA categorization in input and 2b-IPed sRNA libraries. **C** Proportion of different size classes of mRNA-derived sRNAs in input and 2b-IPed samples. **D** Scatterplot of sRNA accumulation (log10 of RPMs) for individual miRNA families. Elements that accumulate 2-fold or higher sRNAs in 2b-IP samples are highlighted in blue while elements that accumulate 2-fold or higher in input samples are highlighted in red. **E** Violin plot of RNA expression (log10 RPKM) for mRNAs that accumulate sRNAs more than 2-fold in 2b IPed samples (left panel), more than 2-fold in input samples (center panel), and the rest of mRNAs (right panel). *p* values are indicated in the comparisons and indicate the result of a paired *t* test with 2 tails. **F** Size distribution of the vsiRNAs loaded into 2b or input samples. **G** Proportion of 5′ nucleotide distribution in vsiRNAs loaded in 2b or input samples. Two bioreplicates from 2b-IPed sRNA libraries were generated and analyzed

CMV VSR, the 2b protein, interacts with the RNA silencing pathways by direct loading of vsiRNAs [20, 21]. Overexpression of the 2b protein inhibits siRNA-mediated TGS and PTGS silencing [41–44]. Indeed, we generated 2b transgenic plants which confirmed that its overexpression induced a myriad of developmental defects resembling the effects of mutations in key components of the RNA silencing pathways (Fig. 3A). To understand in detail how the 2b protein might interact with RNA silencing pathways within the context of CMV infection, we performed an IP of the 2b protein using commercial antibodies available (see the “Materials and methods” section for details) followed by generation of sRNA high-throughput libraries from naturally infected tissues. The analysis of the sRNA libraries generated (Additional file 1: Table S1), indicated that 2b can bind to all sRNAs categories, with a preference for mRNA-derived sRNAs (1.6-fold increase in 2b-IP samples and 25.2% of all 2b-loaded sRNAs, Fig. 3B, C). 2b was also able to load miRNA (Additional file 2: Fig S6), TE-derived sRNAs, and intergenic-derived sRNAs, although these last ones were relatively depleted from the IP (2-fold depletion compared to input samples, Fig. 3B). 2b-loaded mRNA-derived sRNAs corresponded to 2211 mRNAs (5.4% of all mRNAs, sRNA accumulating equal, or more than 2-fold with a *p* value inferior to 0.05, Fig. 3D). We further enquired the potential role of these sRNAs during infection by analyzing mRNA expression under CMV infection, which has been studied previously [45, 46]. Our analysis of public RNA sequencing data [45] indicated that mRNAs that produced sRNAs preferentially incorporated into 2b, reduced their expression significantly during infection compared to mRNAs that did not incorporate sRNAs into 2b (Fig. 3E). This indicated that the loading of mRNA-derived sRNAs into 2b could be involved with their transcriptional regulation during CMV infection.

vsiRNAs followed mRNA-derived sRNAs as preferentially loaded sRNAs (25.2% of all 2b-loaded sRNAs, Fig. 3B and Additional file 2: Fig S6). 2b-loaded vsiRNAs are of 20- and 21-nt, similar to the overall vsiRNAs present in input samples (Fig. 3B–F and Fig. 2E). This indicated that 2b-loaded vsiRNAs are an important part of the overall vsiRNA population accumulating during infection. Contrary to AGO proteins, 2b did not present a clear 5′ nucleotide preference in the vsiRNAs that it is capable of loading (Fig. 3G). Thus, CMV VSR, 2b, had an active role during viral infection both loading mRNA-derived sRNAs, with a potential transcriptional regulation role, and vsiRNAs.

### vsiRNAs target endogenous genes associated with photosynthesis, generation of metabolites, and translation

**Fig. 4:**
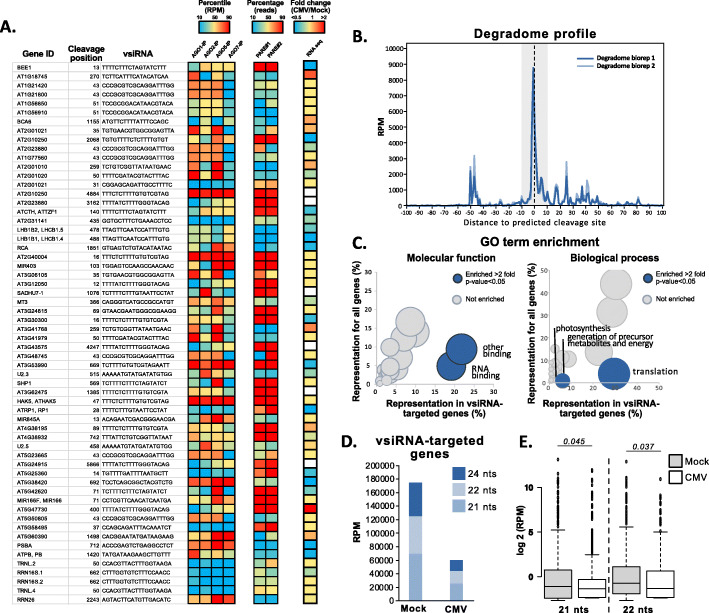
Analysis of vsiRNA-targeted RNAs. **A** Table of the genes targeted by vsiRNAs showing their gene ID, cleavage position, vsiRNA targeting, the level of their presence in different AGO IPs (showed as a heat map for their RPM accumulation), the accumulation of PARE reads in the predicted cleavage position (showed as a heat map for the percentage of reads from the specific mRNA that map to the cleavage position), and the fold change of mRNA expression under CMV infection (showed as a heat map of the fold change of mRNA expression in CMV vs mock). **B** PARE read profile relative to the predicted cleavage position identified by PAREsnip. **C** Analysis of GO term enrichment. Left panel shows the analysis according to molecular function and right panel shows the analysis for biological process. Blue-colored dots show categories enriched equal or more than 2-fold with a *p* value inferior to 0.05. **D** Proportion of 21-, 22-, and 24-nt sRNAs derived from vsiRNA-targeted genes in mock and CMV-infected sRNA libraries. **E** Box-plot representing the accumulation (log2 RPM) of 21- and 22-nt sRNAs derived from individual mRNAs in mock and CMV-infected sRNA libraries. Whiskers extend to data points that are less than 1.5 x IQR away from 1st/3rd quartile. *p* values are indicated in the comparisons and indicate the result of a paired *t* test with 2 tails. Two bioreplicates from PARE RNA libraries were generated and analyzed

Our data indicated that vsiRNAs were incorporated into all the AGOs analyzed, representing a substantial portion of the immunoprecipitated sRNAs. To further understand if these vsiRNAs could be actively incorporated into the endogenous RNA silencing machinery and target host mRNAs, we performed PARE sequencing of CMV-inoculated tissues (Figs. 1A and 4A, B). The analysis of these libraries using PAREsnip [47] identified 61 endogenous mRNAs with evidences of vsiRNA-induced cleavage (targets confirmed in both bioreplicates using astringent PAREsnip parameters, Fig. 4A, B and Additional file 1: Table S5). vsiRNAs that were predicted to target genes were loaded in all the AGOs analyzed with a slight preference for AGO5 loading (Fig. 4A-left column and Additional file 2: Fig S7). As expected from real vsiRNA targets, these mRNAs presented a read peak mapping to the predicted cleavage position of the vsiRNA in the mRNA sequenced (Fig. 4B), which in some cases accounted to up the majority of PARE reads mapping to the predicted target (Fig. 4A, middle column). Together with this, some vsiRNA-targeted mRNAs reduced their accumulation during CMV infection in RNA sequencing datasets from public repositories [45], pointing to an effect in the accumulation of the targeted mRNAs (Fig. 4A, right column).

To further characterize the vsiRNA-targeted mRNAs, we analyzed the Gene Ontology (GO) term enrichment according to molecular function. Interestingly, vsiRNA-targeted mRNAs had an enrichment (>2 fold and *p* value<0.05 calculated through a Fisher’s exact test) in GO categories related to nucleic acid binding, such as RNA binding and other binding (Fig. 4C left panel). The same analysis performed for GO terms according to biological function highlighted a significant enrichment (>2 fold and *p* value<0.05 calculated through a Fisher’s exact test) in genes associated with photosynthesis, translation, and generation of precursor metabolites and energy (Fig. 4C right panel). These target genes included two photosystem II light harvesting complex LHCB1.4 and LHCB1.5, and the tandem zinc finger protein (ATCTH), all well-characterized mediators of the photosynthesis and translation processes, respectively (Fig. 4A) [48–50]. Interestingly, CMV-derived sRNAs have been previously associated with the downregulation of photosynthesis associated genes in *N. benthamiana* [27, 51]

Finally, we analyzed if vsiRNA-mediated cleavage induced the initiation of the RNA silencing amplification of their mRNA targets [52]. To that end, we analyzed the proportion of 21-, 22-, and 24-nt mRNA-derived sRNAs in mock and CMV-infected high-throughput sRNA libraries (Fig. 4D). In general, vsiRNA-targeted mRNAs did not produce higher levels of sRNAs, as it would be expected of a canonical secondary siRNA biogenesis pathway (Fig. 4D). Indeed, 21- and 22-nt mRNA-derived sRNAs from vsiRNA targets are significantly decreased in CMV-infected sRNA libraries (Fig. 4E). In sum, vsiRNAs were incorporated into the canonical RNA silencing pathways and were active regulators of endogenous mRNAs.

### Validation of vsiRNA biological activity

**Fig. 5:**
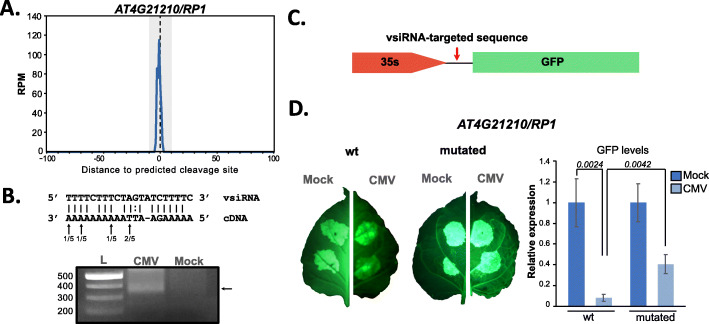
Validation of vsiRNA biological activity. **A** PARE read profile relative to the predicted cleavage position identified by PAREsnip for AT4G21210 (*RP1*). **B** Alignment of the vsiRNA to the predicted target position for AT4G21210 (*RP1*). Cleavage positions identified by 5′ RACE are indicated with arrows, with the number of clones identified at each specific position relative to the total number of clones analyzed indicated. A PCR-amplified 5′ RACE product for AT4G21210 (*RP1*) specific of CMV-infected tissues is shown in the image. **C** Diagram of the design for the constructs used for transient expression analysis. **D** Transient expression of the target sequence of AT4G21210 in *N. benthamiana* showed by representative pictures of the intensity of the GFP in mock and infected leaves and their expression levels measured by RT-qPCR. Error bars depict standard deviation normalized to the average mock values, and *p* values are indicated in the comparisons and indicate the result of an unpaired *t* test with 2 tails

As a proof of concept on the ability of vsiRNAs to target mRNAs, we selected two candidate genes: AT4G21210 (*RP1*) a chloroplastic pyruvate orthophosphate dikinase (PPDK) regulatory protein with a known role in photosynthesis [53, 54], and AT4G36195, a serine carboxypeptidase S28 family protein (Figs. 4A and 5A and Additional file 2: Fig S8A). These two genes were targeted by two vsiRNAs that accumulate preferentially in AGO2 and AGO1, respectively (Additional file 2: Fig S8C and D), and had a clear PARE read peak at their predicted cleavage position (Fig. 5A and Additional file 2: Fig S8A). In accordance with our preliminary analysis, the expression of both genes (analyzed by RT-qPCR) was significantly downregulated in plants infected with CMV (Additional file 2: Fig S8E). Next, to determine that this downregulation takes place specifically due to the activity of vsiRNAs, we performed a transient expression assay in *N. benthamiana* (Fig. 5D and Additional file 2: Fig S8B). To that aim, we generated two constructs each one containing the vsiRNA-targeted sequence from the two selected candidate genes, driven by the 35S promoter and located in the 5′UTR of the GFP sequence, which allowed us to both visualize the expression of this transgene and to quantify its accumulation levels (Fig. 5C). These constructs were then agroinfiltrated in both mock and 7 dpi CMV-infected *N. benthamiana* leaves, and the level of accumulation of GFP transcripts was monitored by RT-qPCR (Fig. 5D and Additional file 2: Fig S8B). These analyses indicated that the expression of both constructs was significantly downregulated in the infected plants indicating that the downregulation of both genes is associated with the presence of the region targeted by vsiRNAs (Fig. 5D and Additional file 2: Fig S8B). Furthermore, this downregulation was partially alleviated in both constructs when the vsiRNA-targeted site was mutated in 4 positions [55] to affect the binding of the vsiRNAs (Fig. 5D and Additional file 2: Fig S8B). Nevertheless, the introduction of these mutations did not return expression of the construct to wild type levels, which might be due to vsiRNAs still retaining some binding ability to the target site, as observed in Liu et al. (2014) when comparing 4, 5, and 6 mismatches in the 5′ seed of a miRNA binding site [55]. We additionally confirmed the targeting of *RP1* by its corresponding vsiRNA by modified 5’RACE [56], which confirmed the targeting position identified by PARE sequencing (Fig. 5B). In summary, these results proved the ability of vsiRNAs to target plant genes and effectively decrease their expression.

## Discussion

RNA silencing plays a fundamental role in the defense response to viruses due to their obligate intracellular nature [57]. Even though the characteristics of this pathway have been widely studied since it was first described [58, 59], there are still some aspects that remain poorly understood. Here, we have studied a less known aspect of this pathway, the overall interference with the endogenous RNA silencing machinery, via the sequencing of viral and endogenous sRNAs, the analysis of vsiRNA incorporation into different AGO proteins and the identification of endogenous mRNAs targeted by vsiRNAs. Moreover, we have characterized the sRNAs bound to the VSR of CMV, the 2b protein. Altogether, our data provides a complete portrait of this complex plant-pathogen interaction.

The effects of CMV infection on the endogenous sRNA populations had been previously described only for the VSR-deficient mutant of CMV and CMV-∆2b, which induces the production of RDR1-dependent virus-activated siRNAs (vasiRNAs) that target thousands of endogenous loci [36]. Other previous studies focused on the characterization of the vsiRNAs generated from the genome of CMV [29, 60] or its satellite RNA [61, 62], similar to studies in other plant viruses and pathogenic RNAs [29, 31, 63, 64]. In our work, we have studied the changes experienced by the endogenous *Arabidopsis* sRNA populations under the infection by the CMV strain *Fny*, which is symptomatic in *Arabidopsis* and other hosts [65–67]. We observed that endogenous sRNA populations were strongly affected by the massive accumulation of vsiRNAs, which accounted to more than 43% of all the sRNA reads in infected tissues. Although the level of accumulation of viral genomes per cell for CMV is unknown (a parameter known as cellular multiplicity of infection or MOI), viruses like *Tobacco etch virus* (TEV) [68], *Tobacco mosaic virus* (TMV) [69], or *Turnip mosaic virus* (TuMV) [70] accumulate 1 to 2 genomes per infected cell at later infection times, with up to 6 genomes per cell [69] and up to 42% of infected cells [68] at earlier infection times (10 dpi). Our tissues correspond to later infection times (30 dpi) although we did not observe a reduction in the accumulation of viral RNAs by Northern blot (Additional file 2: Fig S1C). Interestingly, CMV vsiRNA accumulation was not correlated with the accumulation of CMV genomic RNAs. This disconnection between viral genomic RNA and vsiRNA accumulation is probably explained by the RDR-mediated amplification of vsiRNAs [14, 32]. The massive amount of vsiRNAs might not only accumulate at infected cells, since the antiviral RNA silencing signal is known to spread both locally [71] and systematically [72] providing immunization of tissues before viral infection.

Our work exemplifies the consequences of using RNA silencing as the main mechanism to provide antiviral immunization in plants. We show that massive vsiRNA accumulation caused a decrease in the presence of almost all sRNA categories. This increased presence of vsiRNA was not only a consequence of their great accumulation in the cytoplasm, since we also identified their loading into the different AGO proteins studied here (AGO1, AGO2, AGO5, and AGO7) indicating their active incorporation into effectors of RNA silencing. Interestingly, all the AGOs studied here showed a similar percentage of vsiRNAs (with AGO7, which has a positive effect against CMV infection, being the AGO protein where vsiRNAs occupied a higher percentage of the loaded sRNAs). AGO proteins prefer loading sRNAs with certain 5′ nucleotides [37], but they reflect the population of sRNAs present in their main subcellular niche. For example, AGO1 loads increased amount of epigenetically active siRNAs (easiRNAs) from TEs when heterochromatin is compromised in DECREASE IN DNA METHYLATION 1 (DDM1) mutants [26], or in other examples where the cell is loaded with anomalous proportions of exogenous sRNAs [4, 73]. Our work reveals that this is the case for probably all AGO proteins. Additionally, our data indicates that the antiviral roles of AGO1 and AGO2 are probably influenced by their increased accumulation during CMV infection compared to other AGOs (such as AGO5 or AGO7). Epigenetic changes in the AGO2 promoter region are associated with its increased expression under CMV infection in benzo-(1,2,3)-thiadiazole-7-carbothioic acid *S*-methyl ester (BTH)-primed plants [74]. It is plausible to speculate that the promoter of antiviral (or stress responsive) AGOs are prone to experience epigenetic changes or harbor transcription factor binding sites that enhance their expression during stress. CMV is indeed connected to epigenetic changes through the targeting of 24-nt sRNA characteristic of the RdDM pathway by its VSR 2b [20, 42]. A reduced amount of 24-nt sRNAs will cause a reduction in DNA methylation that might affect the transcription of epigenetically labile regions [75], such as the ones present in the promoter region of AGO2. In line with these potential epigenetic changes, we detected that selected TEs enriched in all contexts of DNA methylation and the repressive histone mark H3K9me2, produced higher levels of 21-nt sRNAs from their transcripts. Furthermore, this increased production of 21-nt sRNAs is associated with the presence of 2b in the genome of CMV. It is possible that the production of 21-nt sRNAs from TEs reflects epigenetic changes (both at the DNA methylation and histone levels) under CMV infection, which is known to induce changes in the DNA methylation levels of its host as previously shown in *Nicotiana benthamiana* [76].

Together with selected TEs, exceptionally, some miRNA families and mRNAs also accumulated higher levels of 21-nt sRNAs. Increased presence of miRNAs might be associated with their loading into AGOs other than AGO1 or the VSR 2b, since we detected an enrichment of certain miRNA families under CMV infection in AGO2-, AGO5-, and 2b-IPed sRNA libraries. It is plausible that AGO2 and AGO5 loading of miRNAs might be a mechanism that compensates the decrease of miRNAs loaded into AGO1. The disruption of the mock profile of miRNAs could be impacting the normal development in CMV-infected plants, as this class of sRNAs play an important role in the regulation of plant development [77]. Indeed, CMV-induced developmental symptoms are conditioned partly by 2b-AGO1 interactions and the consequent interference with miRNA regulated gene expression [43].

To complement the overview of the interaction of CMV with RNA silencing pathways, we further immunoprecipitated and sequenced sRNAs associated with the VSR of CMV, 2b. The previous analysis of the sRNAs associated with 2b identified an enrichment of 24 nt sRNAs [20]. In our study, we could not identify any sRNA size class with a preferential enrichment, although we found an enrichment of mRNA-derived siRNAs, pointing to the technical success of the technique. We think that these differences might account for the use of a commercial antibody in our study and also our analysis within the context of a CMV *Fny* infection, which differs from the wild type scenario of the Hamera et al. (2011) study. mRNA-derived siRNAs enriched in 2b are associated with transcripts that decrease their accumulation significantly during CMV infection, pointing to a role of these sRNAs in the regulation of the expression of the genes from which they originate. sRNAs in other species can promote the stability of their target mRNAs [78] and certain mRNAs experiencing translation inhibition mediated by miRNAs decrease their accumulation to enhance the production of their corresponding protein in microRNA action-deficient (MAD) mutants [79]. Further analysis of the connection between transcriptional and translation effects of viral infection are needed to understand the extend of the role of 2b-sequestered mRNA-derived sRNAs.

Finally, we studied another side effect of antiviral RNA silencing, the ability of vsiRNAs to target endogenous genes. Incorporation of CMV-derived vsiRNAs into RISC complexes indicated that they could potentially target endogenous transcripts. This side effect has been analyzed for several pathogen/parasite-host interaction, where pathogen/parasite-derived sRNAs can target and downregulate the expression of a host gene, in some cases associated with the development of symptoms [22–25, 27]. Our genome-wide analysis allowed us to confirm the targeting of 61 genes by vsiRNAs. Interestingly, CMV vsiRNA targets are enriched in photosynthesis-associated genes (exemplified in our proof-of-concept analysis of *RP1* downregulation) similar to the previous targeting examples in *N. benthamiana* [27, 51], the predicted targets for CaMV vsiRNAs [80], and the target of a sRNA derived from a chloroplast-replicating viroid [81, 82]. These four virus/viroid-host interactions are associated with the mosaic symptomatology, which affects chlorophyll accumulation. Variegated mutants in *Arabidopsis* accumulate reactive oxygen species (ROS) [83], which induces cell death [84] and restrict viral infection [85], a, in principle, disadvantageous strategy for viral progression. A recent report indicates that CMV induces autophagy in *Arabidopsis* and that this response increases both the plant and virus fitness, allowing seed transmission of CMV [86]. vsiRNAs, through the targeting of photosynthesis-associated genes might be a key step in the initiation of anti/proviral autophagy. Alternatively, targeting of photosynthetic factors might be beneficial for the virus by generating a stress response in the plant that accelerates flowering and seed production [87, 88] promoting, indirectly, CMV transmission to the next generation.

## Conclusions

**Fig. 6:**
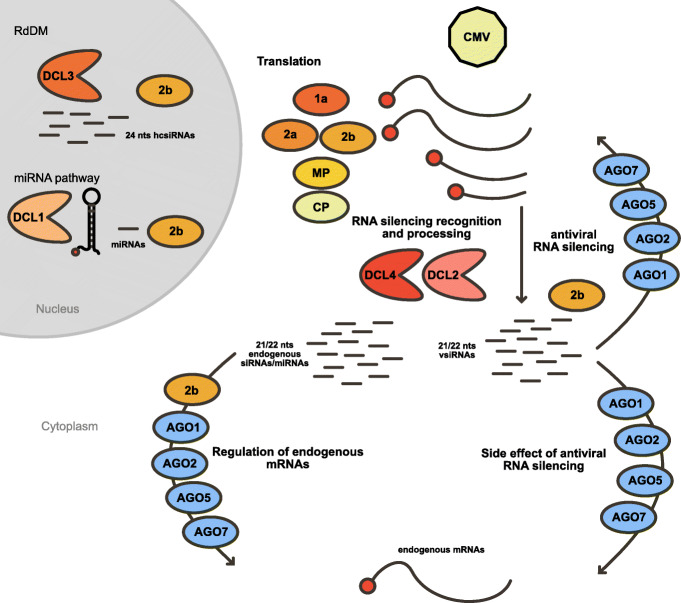
Proposed model. CMV infection leads to the accumulation of its genomic RNAs which are recognized by the RNA silencing machinery producing 21- (mainly) and 22-nt vsiRNAs. These vsiRNAs are then incorporated into the endogenous antiviral machinery mediated by AGO2, AGO1, AGO5, and AGO7 and inhibited by the 2b protein. As a side effect of this antiviral activity, vsiRNAs are also incorporated into the endogenous RNA silencing pathways mediating the silencing of endogenous genes. Together with this, 2b binds to endogenous mRNA-derived sRNAs and is involved in the transcriptional regulation of these genes

Together, our data suggests that vsiRNAs interfere with the endogenous RNA silencing machinery at different levels (Fig. 6): monopolizing the sRNA profile, getting incorporated into all AGO proteins, and targeting endogenous genes. Our results highlight the complex relationship of RNA viruses with the endogenous RNA silencing machinery and the multiple aspects of virus resistance and virulence that this interaction induces.

## Methods

### Plant material and viral inoculations

Two leaves per Col-0 plant in the 4 rosettes leaves stage, 1.04 stage from Boyes et. al 2001 [89] were rub-inoculated with 0.1 M Na_2_HPO_4_ containing the three RNAs that constitutes CMV (strain *Fny*) genome. These RNAs were previously obtained by in vitro transcription using the MAXIscript T7 Transcription Kit (ThermoFisher) and plasmids containing the individual genomic RNA sequences as the template for the transcription. Mock plants were rub-inoculated with Na_2_HPO_4_ buffer. Rosette leaves from these Col-0 plants were taken at 30 dpi to perform sRNA-seq and PARE-seq. At the same time, *N. benthamiana* plants were infected with the same in vitro transcription products to obtain a reservoir of infected tissue, which was used to infect the *ago* mutants for RT-qPCR analysis and the Col-0 and AGO7-HA for IP-sRNA-seq. For all the different analysis, samples were taken 30 dpi and plants were grown in the same chamber under the same conditions (long day, 22^o^C, and 45% humidity).

### Total RNA, sRNA Northern blot AGO immunoprecipitation, sRNA/PARE library construction, and modified 5′RACE

Total RNA was isolated using TRIzol reagent (Invitrogen). For sRNA Northern blot detection, 20 μg of total RNA was loaded in each lane. sRNA gel electrophoresis, blotting, and cross-linking were performed as described in Pall et al. (2008) [90]. Viral genomic RNAs were detected as described in Rio et al. (2015) [91] using the chemical crosslinking strategy described in Pall et al. (2008) [90]. 5 μg of total RNA was loaded in each lane. Probes for the detection of + strand viral genomic RNAs were generated using T3 transcription from the DNA fragments produced using the primers indicated in Additional file 1: Table S6. Probes for the detection of - strand vsiRNAs were generated using T7 transcription from the DNA fragments produced using the primers indicated in Supplementary Table 6 and chemically fragmented as indicated in Martinez (2017) [92]. sRNA libraries were constructed using the NEBNext Small RNA Library Prep Set for Illumina kit (New England Biolabs) following the manufacturer instructions. PARE libraries were constructed following the protocol described in Zhai et al. (2014) using mRNA enriched fractions obtained with the NEB Magnetic mRNA Isolation Kit (New England Biolabs). Modified 5′RACE analysis of vsiRNA cleavage positions was performed as described in Llave et al. (2011) [56] with the primers indicated in Additional file 1: Table S6.

### Gene ontology analysis

Gene ontology (GO) analysis was performed using the GO Term Enrichment tool of The Arabidopsis Information Resource (https://www.arabidopsis.org/tools/bulk/go/index.jsp).

### Gene expression analysis (RT-qPCR)

Three biological replicates of each treatment (mock and virus-infected), consisting of pools of 3/4 *Arabidopsis* plants, were used for the gene expression analysis. RNA extraction from these plants was performed using TRIzol Reagent (Invitrogen) following manufacturer instructions. The extracted RNA was treated with DNase I, RNase-free (ThermoFisher Scientific), and cDNA was synthetized using the RevertAid First-Strand cDNA Synthesis Kit (ThermoFisher Scientific) following the manufacturer instructions. Finally, the level of the mRNAs of interested was measured using 5x HOT FIREPol EvaGreen qPCR Mix Plus (ROX) (Solis Biodyne).

### Transient expression in *Nicotiana benthamiana*

Constructs carrying both the wild-type and the mutated vsRNA-targeted sequences were obtained using the destination vector pB7FWG2.0 (Gateway Cloning), which contains the 35S promoter and an N-terminal GFP using the primers listed in Additional file 1: Table S6. The final constructs were transformed into *Agrobacterium tumefaciens* and agroinfiltrated in both mock and 7 dpi CMV-infected *N. benthamiana*. Two days after infiltration, GFP fluorescence was visualized using an UV light lamp and samples for gene expression analysis were taken. For this last purpose, three biological replicates consisting of pools of two leaves were sampled for each treatment (mock or CMV-inoculated). Protocols were followed as described in the previous section (Gene expression analysis (RT-qPCR)).

### AGO and 2b immunoprecipitation (IP)-sRNA sequencing

IPs were performed using AGO1, AGO2, AGO5, HA, and 2b antibodies from Agrisera (references: AS09 527, AS13 2682, AS10 671, AS12 2220, and AS16 3981, respectively) and following the protocol described in McCue et al. (2016) [93]. Then, the total RNA was extracted using TRIzol Reagent (Invitrogen) following the manufacturer instructions and sRNA libraries were prepared using the NEBNext Small RNA Library Prep Set for Illumina kit (New Englands Biolabs). Two bioreplicates of each IP and their corresponding input control were generated and analyzed.

### Western blot

First, the total protein was extracted from independent biological replicates of 20 dpi mock-inoculated and CMV-infected rosettes using a standard extraction buffer (100 mM Tris pH 7.5 + 2% SDS) and quantified using Pierce Coomassie (Bradford) Protein Assay Kit (ThermoFisher Scientific). Then, 30 μg of total protein were run in an SDS-PAGE gel for 2 h at 120V and transfer to a Roti-Fluoro PVDF membrane (ROTH) on a semi-dry transfer for 1 h at 15V. The membrane was then blocked for 2 h on 5% milk in PBS-T solution at 4°C. Afterwards, three washes of 5 min with PBS-T were done. The AGO antibodies used were the same ones used for Immunoprecipitation and the actin antibody was also from Agrisera (AS13 2640). The primary antibodies were incubated overnight at 4°C in the following dilutions: AGO1, AGO2, and actin 1:10.000 and AGO5: 1:5.000, in 5% milk in PBS-T. Afterwards, three washes of 5 min with PBS-T were done. Then, the membrane was incubated for 1.5 h with the secondary antibody (Goat anti-Rabbit IgG (H&L), HRP conjugated, Agrisera, AS09 602), which was diluted 1:10.000 on 5% milk in PBS-T, followed by three washes of 5 min with PBS-T. Finally, the Western blot was revealed using the Amersham ECL Prime Western Blotting Detection Reagent (ROTH) following the manufacturer instructions and using a LAS-3000 Imaging System (Fuji). The intensity of the obtained bands was measure using ImageJ blot analysis tools. Then, the intensity of each AGO band was normalized to the intensity of the corresponding actin band. The normalized value was used to calculate the CMV/Mock ratio of protein accumulation. Two bioreplicates of each Western blot were generated and analyzed.

### Small RNA and PARE library bioinformatic analysis

The resulting sequences were de-multiplexed, adapter trimmed, and filtered on length and quality. Two bioreplicates were sequenced for sRNA analysis. sRNAs were matched to the TAIR 10 version of the *Arabidopsis* genome. Library size was normalized by calculating reads per million of 18–28 nt genome-matched for sRNAs or calculating reads per million for all 19–21-nt genome matched reads for PARE sequencing. sRNA and PARE alignments were performed using bowtie [94] with the following parameters –t –v2 that allows 2 mismatches to the alignments. For sRNA categorization, sRNA libraries were aligned to individual indexes generated for each genomic category. For categorization of sRNAs as mRNA- and intergenic-derived, the sequences matching to miRNAs and TEs, respectively, were subtracted. For PARE library analysis, vsiRNA cleavage events were identified using PARESnip [95]. Identification of high-confidence target sites was performed using astringent PARESnip criteria and considering the presence of such target site in the two bioreplicates. Principal component analysis was performed using the plotPCA tool of deepTools [96] through the Galaxy platform [97]. Two bioreplicates of each sRNA and PARE library were generated and analyzed.

## Supplementary Information

**Additional file 1: Supplementary tables**. **Table S1**. Libraries used in this study. **Table S2**. miRNA accumulation (RPM) in mock and CMV-infected sRNA libraries. **Table S3**. 21-nt mRNA-derived sRNA accumulation (RPM) in mock and CMV-infected sRNA libraries. **Table S4**. 21-nt TE-derived sRNA accumulation (RPM) in mock and CMV-infected sRNA libraries. **Table S5**. vsiRNA-targeted genes identified by PARE sequencing. **Table S6**. Primers used in this study.

**Additional file 2: Supplementary figures. Figure S1**. Origin of CMV-derived vsiRNAs. **Figure S2**. Characterization of endogenous sRNA libraries from mock and CMV-infected tissues. **Figure S3**. Characterization of TEs producing increased 21-nt sRNAs under CMV infection. **Figure S4**. AGO antiviral activity and accumulation during CMV infection. **Figure S5**. Heat map of miRNA accumulation in different AGO-IP sRNA libraries for mock and infected tissues. **Figure S6**. Characteristics of 2b-IPed sRNAs. **Figure S7**. Accumulation in different AGOs of vsiRNA targeting mRNAs identified by PARE sequencing. **Figure S8**. Characteristics of selected vsiRNAs and their targeted genes. **Figure S9**. Uncropped Northern blot for the detection of CMV genomic RNA1. **Figure S10**. Uncropped Northern blot for the detection of CMV genomic RNA2. **Figure S11**. Uncropped Northern blot for the detection of CMV genomic RNA3. **Figure S12**. Uncropped Northern blot for the detection of vsiRNAs derived from CMV genomic RNA1 **Figure S13**. Uncropped Northern blot for the detection of vsiRNAs derived from CMV genomic RNA2. **Figure S14**. Uncropped Northern blot for the detection of vsiRNAs derived from CMV genomic RNA3. **Figure S15**. Uncropped Northern blot for the detection of miR168. **Figure S16**. Uncropped Northern blot for the detection of the snRNA U6. **Figure S17**. Uncropped ethidium bromide-stained agarose gel used as loading control for the detection of CMV genomic RNAs. **Figure S18**. Uncropped ethidium bromide-stained agarose gel used for the analysis and cloning of 5’RACE fragments derived from AT4G21210. **Figure S19**. Uncropped Western blot gels used in the detection and quantification of AGO and Actin proteins.

**Additional file 3.** Review history.

## Data Availability

All raw and processed sequencing data generated in this study have been submitted to the NCBI Gene Expression Omnibus (GSE169677) [98].
