# Supplementary material for: Reprogramming of RNA silencing triggered by cucumber mosaic virus infection in Arabidopsis
Source: Genome Biol. 2021 Dec 15;22:340. doi: 10.1186/s13059-021-02564-z (PMC8672585; doi:10.1186/s13059-021-02564-z)

ADDITIONAL FILE LEGENDS

Additional File 1: Supplementary tables

Table S1. Libraries used in this study.

Table S2. miRNA accumulation (RPM) in mock and CMV-infected sRNA libraries.

Table S3. 21-nt mRNA-derived sRNA accumulation (RPM) in mock and CMV-infected sRNA libraries.

Table S4. 21-nt TE-derived sRNA accumulation (RPM) in mock and CMV-infected sRNA libraries.

Table S5. vsiRNA-targeted genes identified by PARE sequencing.

Table S6. Primers used in this study.

Additional File 2: Supplementary figures

Figure S1. Origin of CMV-derived vsiRNAs. A. Proportion of vsiRNAs according to their genomic RNA origin. **B.** Distribution of vsiRNAs mapped along each of the genomic RNAs from CMV. **C.** Northern blot detection of CMV genomic RNAs and vsiRNAs at 10, 20 and 30 dpi. EtBr stained total RNA was used as a loading control of genomic RNAs while U6 snRNA was used as a loading control of sRNA loading.

Figure S2. Characterization of endogenous sRNA libraries from mock and CMV-infected tissues. A. Principal component analysis for the libraries analyzed. **B-G.** sRNA size distribution for mock and CMV-infected libraries for different categories: miRNAs (**B.**), mRNAs (**C.**), TEs (**D.**), Intergenic (**E.**), tRNA (**F.**) and rRNA (**G.**).

Figure S3. Characterization of TEs producing increased 21-nt sRNAs under CMV infection. A. TE categorization for all TEs in the Arabidopsis genome and TEs that produce increased amounts of 21-nt sRNAs. **B.** DNA methylation values at each specific context (CG, CHG and CHH) for all TEs in the *Arabidopsis* genome (white boxes) and TEs that produce increased amounts of 21-nt sRNAs (grey boxes). **C-D.** Log 2 values of size (**C**) and H3K9me2 content (**D**) for all TEs in the *Arabidopsis* genome (white boxes) and TEs that produce increased amounts of 21-nt sRNAs (grey boxes). **E.** Log10 value of the ratio of 21-nt sRNAs produced by TEs with increased production of sRNAs under CMV infection in CMV-infected plants (white box) and CMV-Δ2b-infected plants (grey box). All box plots whiskers extend to 5th and 95th percentile. p-values are indicated in the comparisons and indicate the result of a paired t-test with 2 tails.

Figure S4. AGO antiviral activity and accumulation during CMV infection. A. Analysis of sensitivity to CMV infection of the AGO mutants for the proteins analyzed in this work (AGO1, AGO2, AGO5 and AGO7). Sensitivity to viral infection was quantified as the rosette radius of CMV-infected mutant compared to mock-infected plants. **B.** Analysis of the relative intensity of AGO accumulation detected by Western blot. Error bars represent the standard deviation between two bioreplicates of each Western blot. All box plots whiskers extend to 5th and 95th percentile. p-values are indicated in the comparisons and indicate the result of a paired t-test with 2 tails.

Figure S5. Heat map of miRNA accumulation in different AGO-IP sRNA libraries for mock and infected tissues.

Figure S6. Characteristics of 2b-IPed sRNAs. A. Proportions of endogenous siRNAs and vsiRNAs of different size classes for input and 2b-IP sRNA libraries. **B.** Heat map of miRNA accumulation in 2b-IP and input sRNA libraries.

Figure S7. Accumulation in different AGOs of vsiRNA targeting mRNAs identified by PARE sequencing. All box plots whiskers extend to 5th and 95th percentile. p-values are indicated in the comparisons and indicate the result of a paired t-test with 2 tails.

Figure S8. Characteristics of selected vsiRNAs and their targeted genes. A. PARE read profile relative to the predicted cleavage position identified by PAREsnip for AT4G36195. Alignment of the vsiRNA to the predicted target position for AT4G36195 is shown in the bottom panel. **B.** Transient expression of the target sequence of AT4G36195 in *N. benthamiana* showed by representative pictures of the intensity of the GFP in mock and infected leaves and their expression levels measured by RT-qPCR. Error bars depict standard deviation normalized to the average mock values for three bioreplicates, p-values are indicated in the comparisons and indicate the result of an unpaired t-test with 2 tails. **C-D.** Accumulation of the vsiRNAs targeting AT4G21210 (C.) and AT4G36195 (D.) in different AGOs**. E.** Expression level of AT4G21210 and AT4G36195 in mock and CMV-infected plants measured by RT-qPCR.

Figure S9. Uncropped Northern blot for the detection of CMV genomic RNA1. Genomic RNA1 is indicated with an asterisk. The same membrane was reprobed three times for each genomic RNA.

Figure S10. Uncropped Northern blot for the detection of CMV genomic RNA2. Genomic RNA2 is indicated with an asterisk. The same membrane was reprobed three times for each genomic RNA.

Figure S11. Uncropped Northern blot for the detection of CMV genomic RNA3. Genomic RNA3 is indicated with an asterisk. The same membrane was reprobed three times for each genomic RNA.

Figure S12. Uncropped Northern blot for the detection of vsiRNAs derived from CMV genomic RNA1. Left part of the image corresponds to the gel shown in Supplementary Figure 1C while right part of the image corresponds to the gel shown in

Figure 1B (not shown in the main figure for this specific CMV genomic RNA). The same membrane was reprobed five times for each genomic RNA, miR168 and the snRNA U6.

Figure S13. Uncropped Northern blot for the detection of vsiRNAs derived from CMV genomic RNA2. Left part of the image corresponds to the gel shown in Supplementary Figure 1C while right part of the image corresponds to the gel shown in Figure 1B. The same membrane was reprobed five times for each genomic RNA, miR168 and the snRNA U6.

Figure S14. Uncropped Northern blot for the detection of vsiRNAs derived from CMV genomic RNA3. Left part of the image corresponds to the gel shown in Supplementary Figure 1C while right part of the image corresponds to the gel shown in Figure 1B (not shown in the main figure for this specific CMV genomic RNA). The same membrane was reprobed five times for each genomic RNA, miR168 and the snRNA U6.

Figure S15. Uncropped Northern blot for the detection of miR168. Left part of the image corresponds to the gel shown in Supplementary Figure 1C (not shown in the supplementary figure for this miRNA) while right part of the image corresponds to the gel shown in Figure 1B. The same membrane was reprobed five times for each genomic RNA, miR168 and the snRNA U6.

Figure S16. Uncropped Northern blot for the detection of the snRNA U6. Left part of the image corresponds to the gel shown in Supplementary Figure 1C while right part of the image corresponds to the gel shown in Figure 1B. The same membrane was reprobed five times for each genomic RNA, miR168 and the snRNA U6.

Figure S17. Uncropped ethidium bromide-stained agarose gel used as loading control for the detection of CMV genomic RNAs.

Figure S18. Uncropped ethidium bromide-stained agarose gel used for the analysis and cloning of 5'RACE fragments derived from AT4G21210.

Figure S19. Uncropped Western blot gels used in the detection and quantification of AGO and Actin proteins.

Figure S1

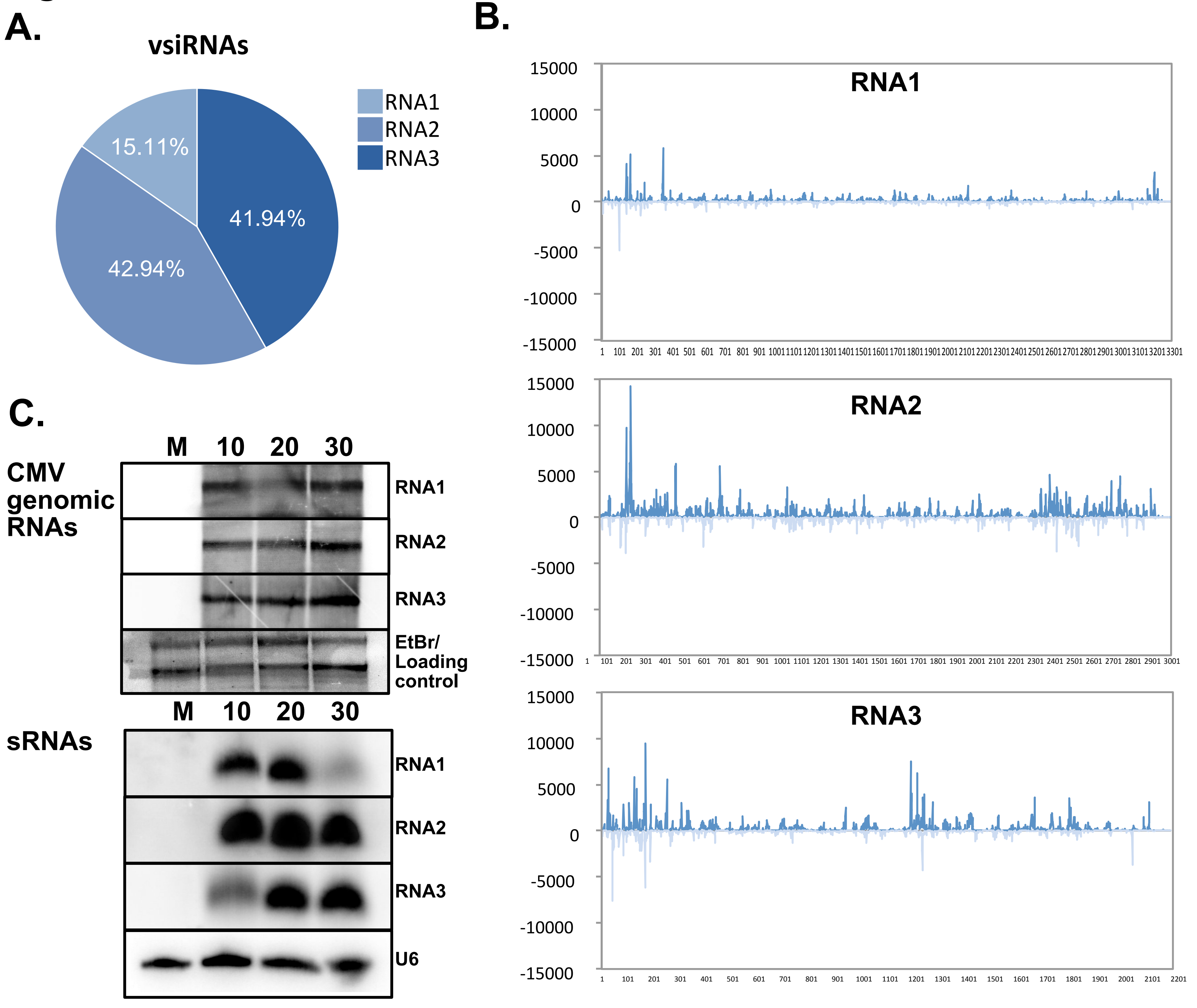
A.
vsiRNAs
RNA1
RNA2
RNA3
15.11%
42.94%
41.94%
B.
RNA1
RNA2
RNA3
15000
10000
5000
0
-5000
-10000
-15000
C.
M
10
20
30
CMV genomic RNAs
RNA1
RNA2
RNA3
EtBr/ Loading control
sRNAs
U6

Figure S2

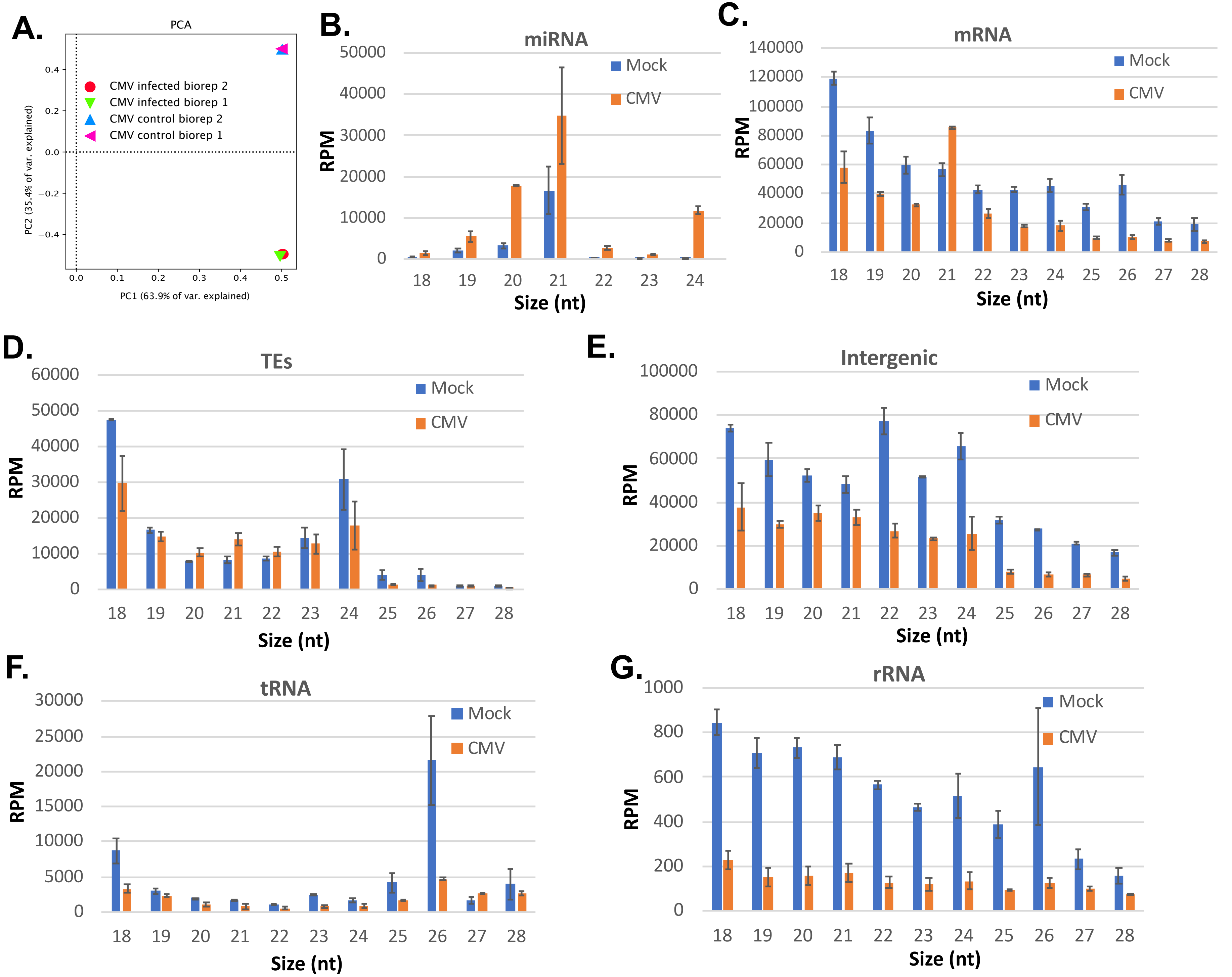
A.
PCA
CMV infected biorep 2
CMV infected biorep 1
CMV control biorep 2
CMV control biorep 1
PC1 (63.9% of var. explained)
PC2 (35.4% of var. explained)
B.
miRNA
Mock
CMV
RPM
Size (nt)
C.
mRNA
Mock
CMV
RPM
Size (nt)
D.
TEs
Mock
CMV
RPM
Size (nt)
E.
Intergenic
Mock
CMV
RPM
Size (nt)
F.
tRNA
Mock
CMV
RPM
Size (nt)
G.
rRNA
Mock
CMV
RPM
Size (nt)

Figure S3

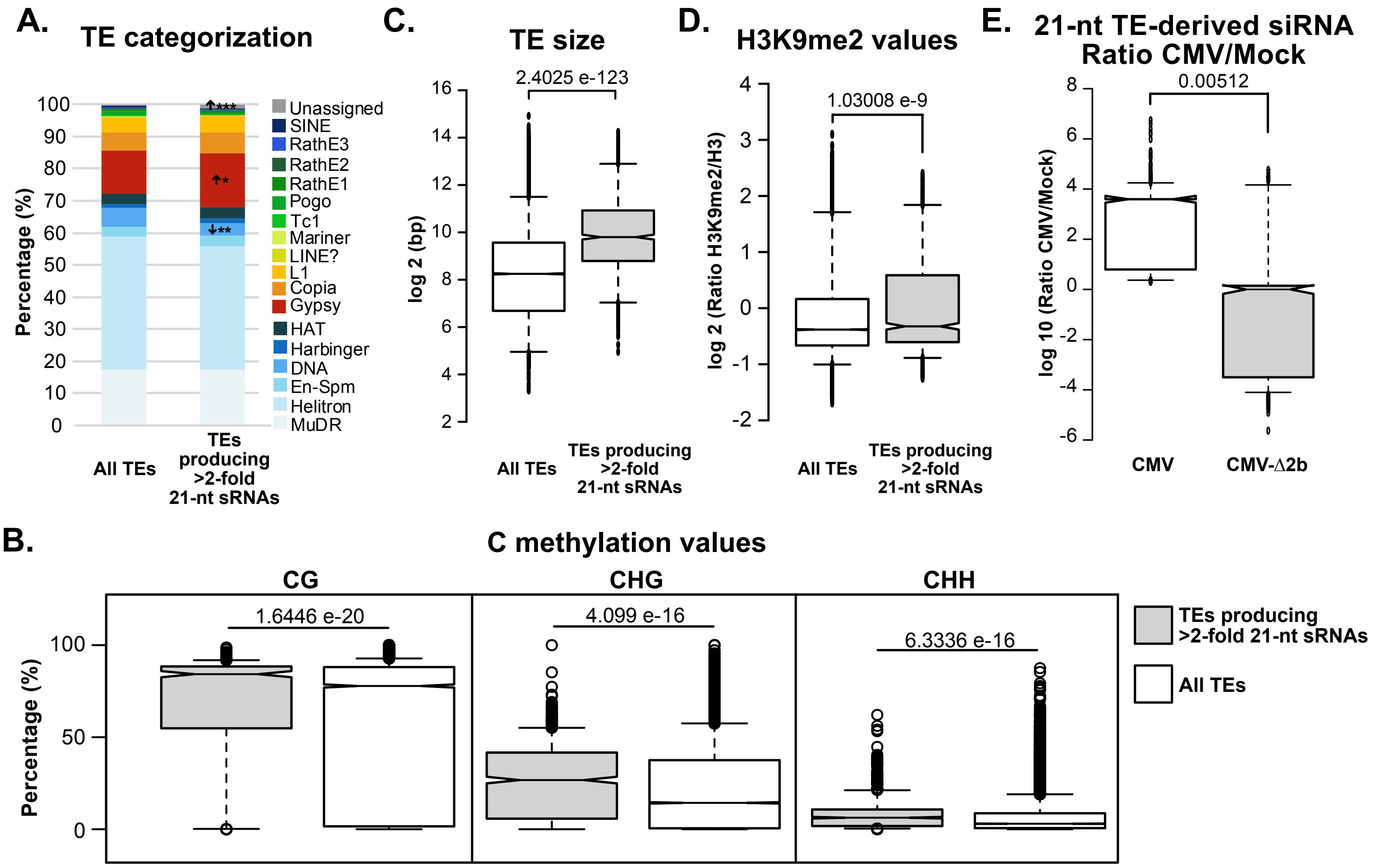

A.
TE categorization
Percentage (%)
100
90
80
70
60
50
40
30
20
10
0
All TEs
TEs producing >2-fold 21-nt sRNAs
↑***
↑*
↓**
Unassigned
SINE
RathE3
RathE2
RathE1
Pogo
Tc1
Mariner
LINE?
L1
Copia
Gypsy
HAT
Harbinger
DNA
En-Spm
Helitron
MuDR
C.
TE size
2.4025 e-123
log 2 (bp)
16
14
12
10
8
6
4
2
All TEs
TEs producing >2-fold 21-nt sRNAs
D.
H3K9me2 values
1.03008 e-9
log 2 (Ratio H3K9me2/H3)
4
3
2
1
0
-1
-2
All TEs
TEs producing >2-fold 21-nt sRNAs
E.
21-nt TE-derived siRNA Ratio CMV/Mock
0.00512
log 10 (Ratio CMV/Mock)
8
6
4
2
0
-2
-4
-6
CMV
CMV-Δ2b
B.
C methylation values
CG
CHG
CHH
1.6446 e-20
4.099 e-16
6.3336 e-16
Percentage (%)
100
50
0
TEs producing >2-fold 21-nt sRNAs
All TEs

Figure S4

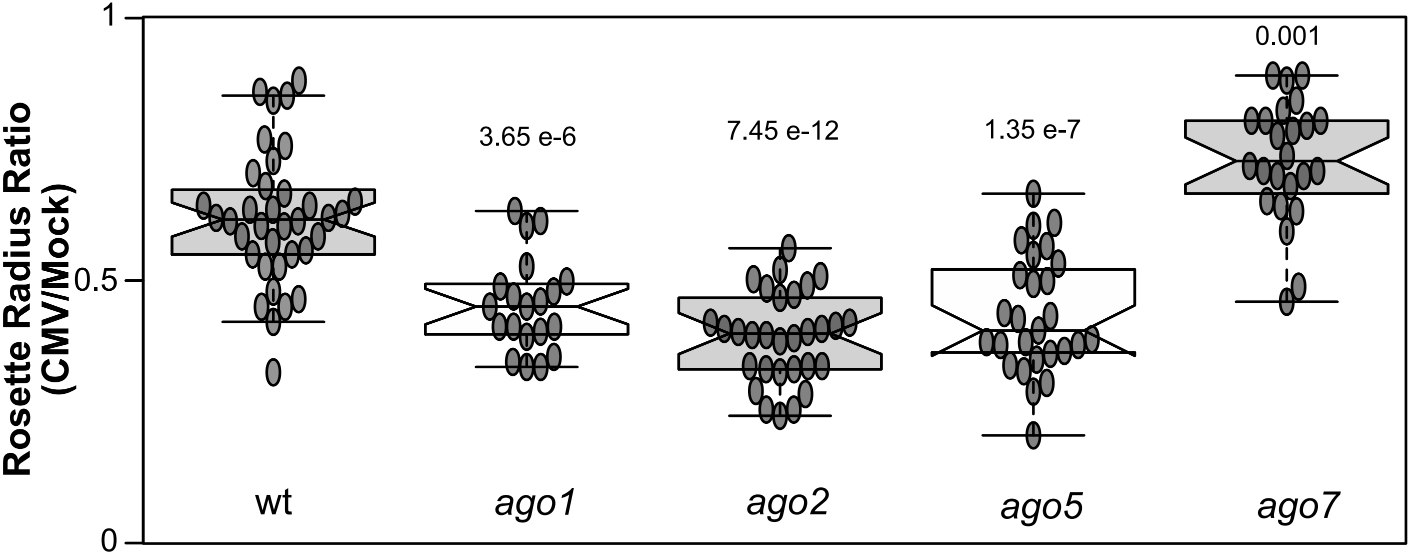
Rosette Radius Ratio
(CMV/Mock)
1
0.5
0
3.65 e-6
7.45 e-12
1.35 e-7
0.001
wt
ago1
ago2
ago5
ago7

B.

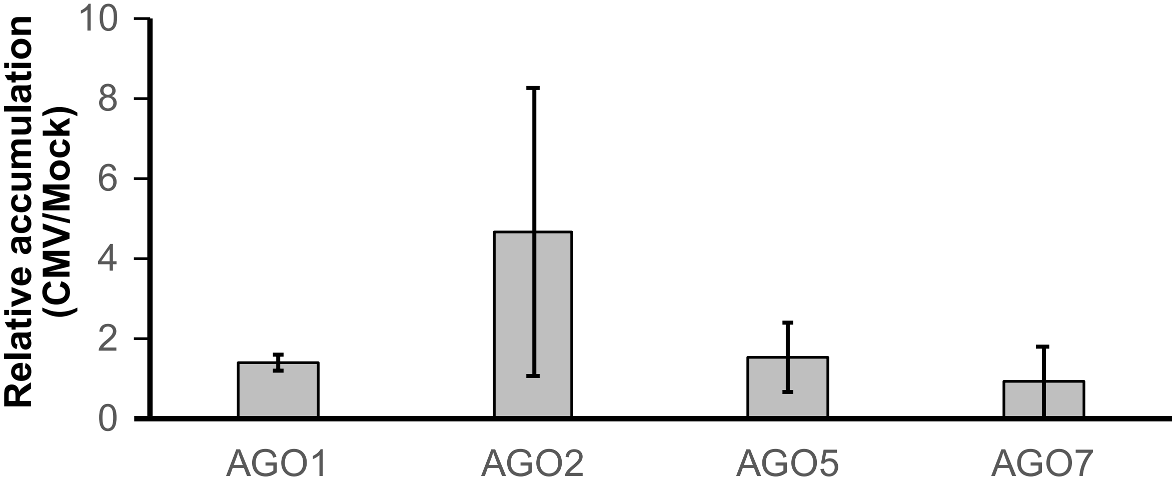
Relative accumulation
(CMV/Mock)
10
8
6
4
2
0
AGO1
AGO2
AGO5
AGO7

Figure S5

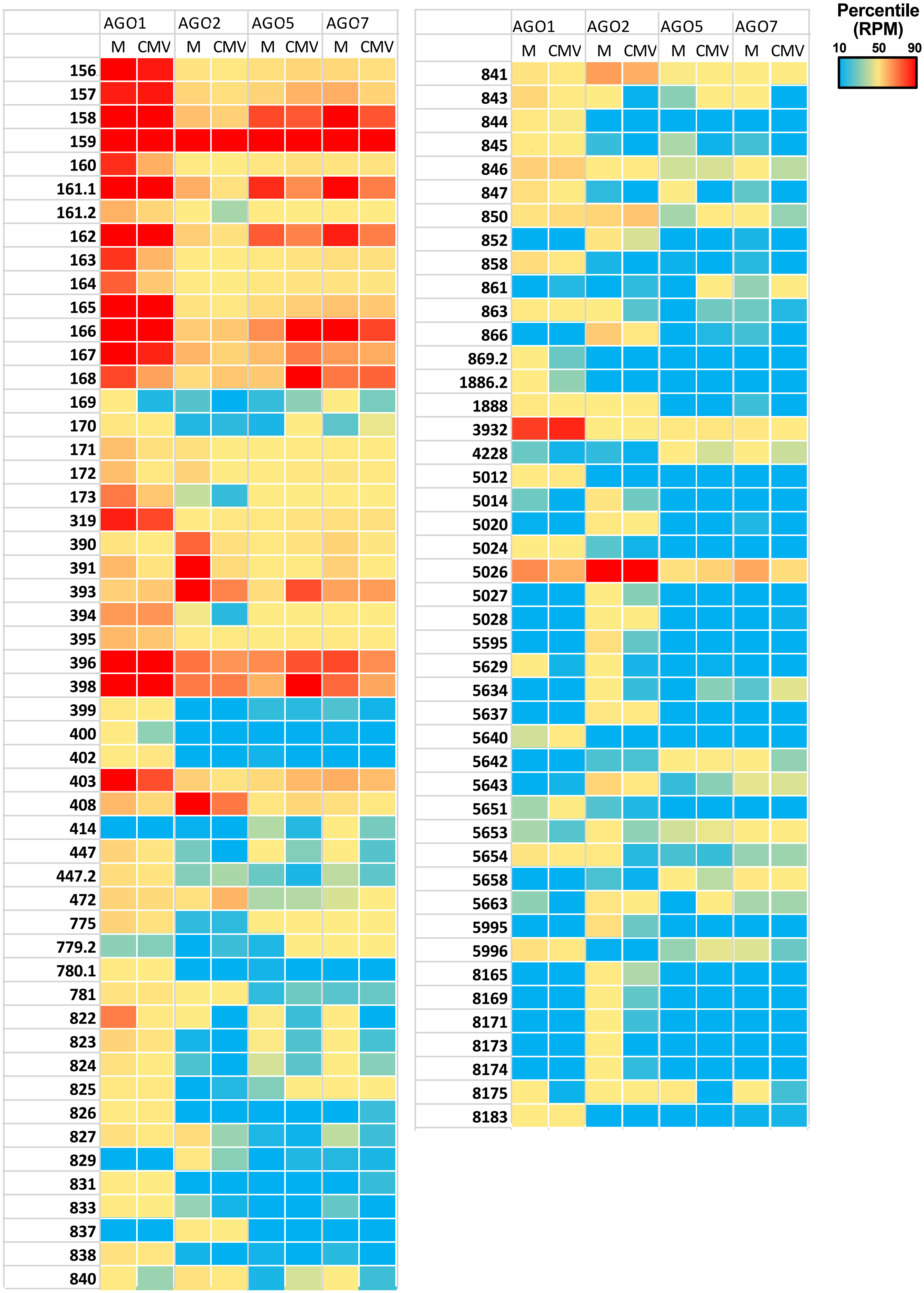
AGO1
AGO2
AGO5
AGO7
M
CMV
156
157
158
159
160
161.1
161.2
162
163
164
165
166
167
168
169
170
171
172
173
319
390
391
393
394
395
396
398
399
400
402
403
408
414
447
447.2
472
775
779.2
780.1
781
822
823
824
825
826
827
829
831
833
837
838
840
841
843
844
845
846
847
850
852
858
861
863
866
869.2
1886.2
1888
3932
4228
5012
5014
5020
5024
5026
5027
5028
5595
5629
5634
5637
5640
5642
5643
5651
5653
5654
5658
5663
5995
5996
8165
8169
8171
8173
8174
8175
8183
Percentile
(RPM)
10
50
90

Figure S6

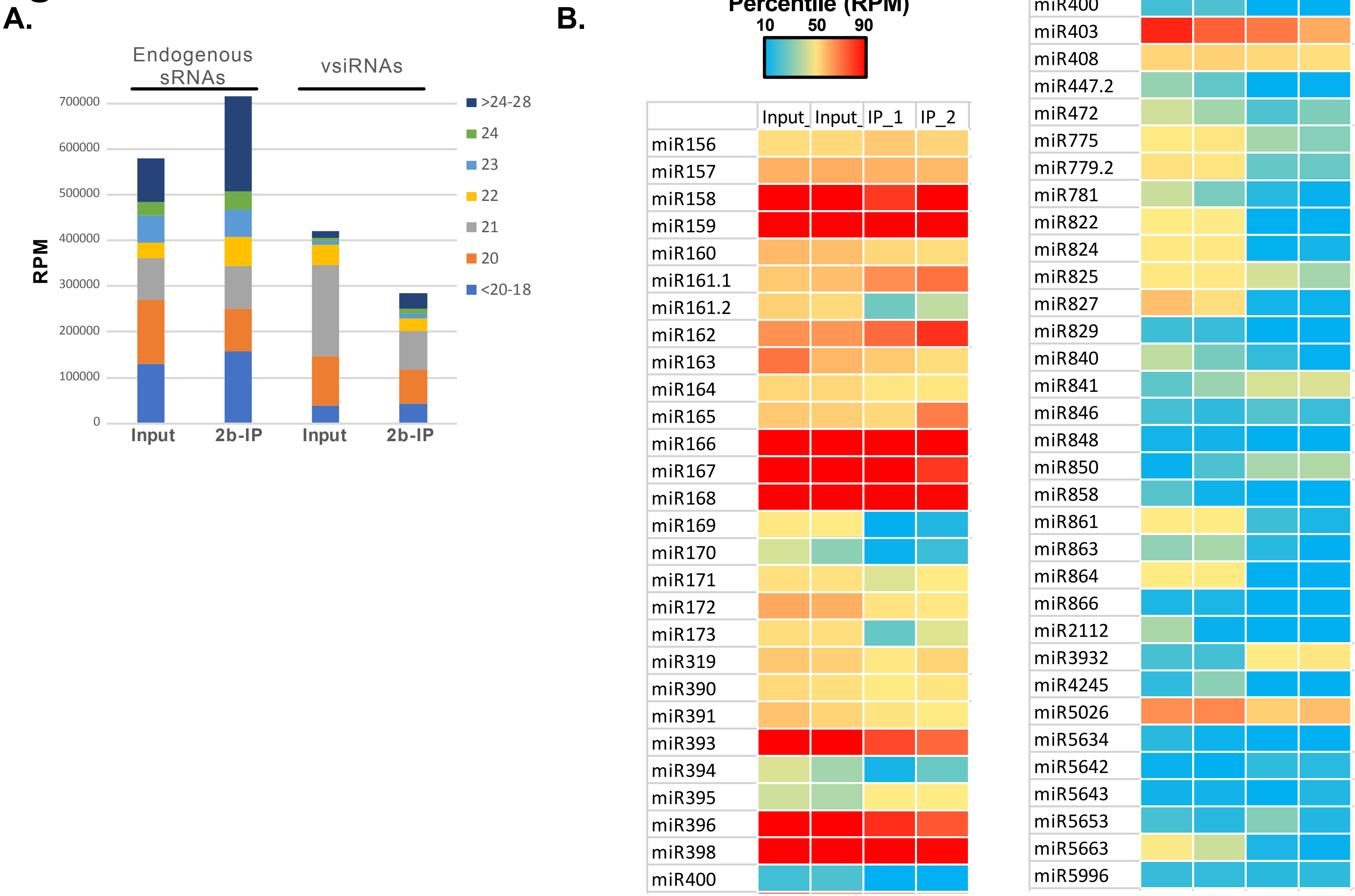
A.
Endogenous sRNAs
vsiRNAs
RPM
700000
600000
500000
400000
300000
200000
100000
0
Input
2b-IP
Input
2b-IP
>24-28
24
23
22
21
20
<20-18
B.
Percentile (RPM)
10
50
90
Input_
Input_
IP_1
IP_2
miR156
miR157
miR158
miR159
miR160
miR161.1
miR161.2
miR162
miR163
miR164
miR165
miR166
miR167
miR168
miR169
miR170
miR171
miR172
miR173
miR319
miR390
miR391
miR393
miR394
miR395
miR396
miR398
miR400
miR400
miR403
miR408
miR447.2
miR472
miR775
miR779.2
miR781
miR822
miR824
miR825
miR827
miR829
miR840
miR841
miR846
miR848
miR850
miR858
miR861
miR863
miR864
miR866
miR2112
miR3932
miR4245
miR5026
miR5634
miR5642
miR5643
miR5653
miR5663
miR5996

Figure S7

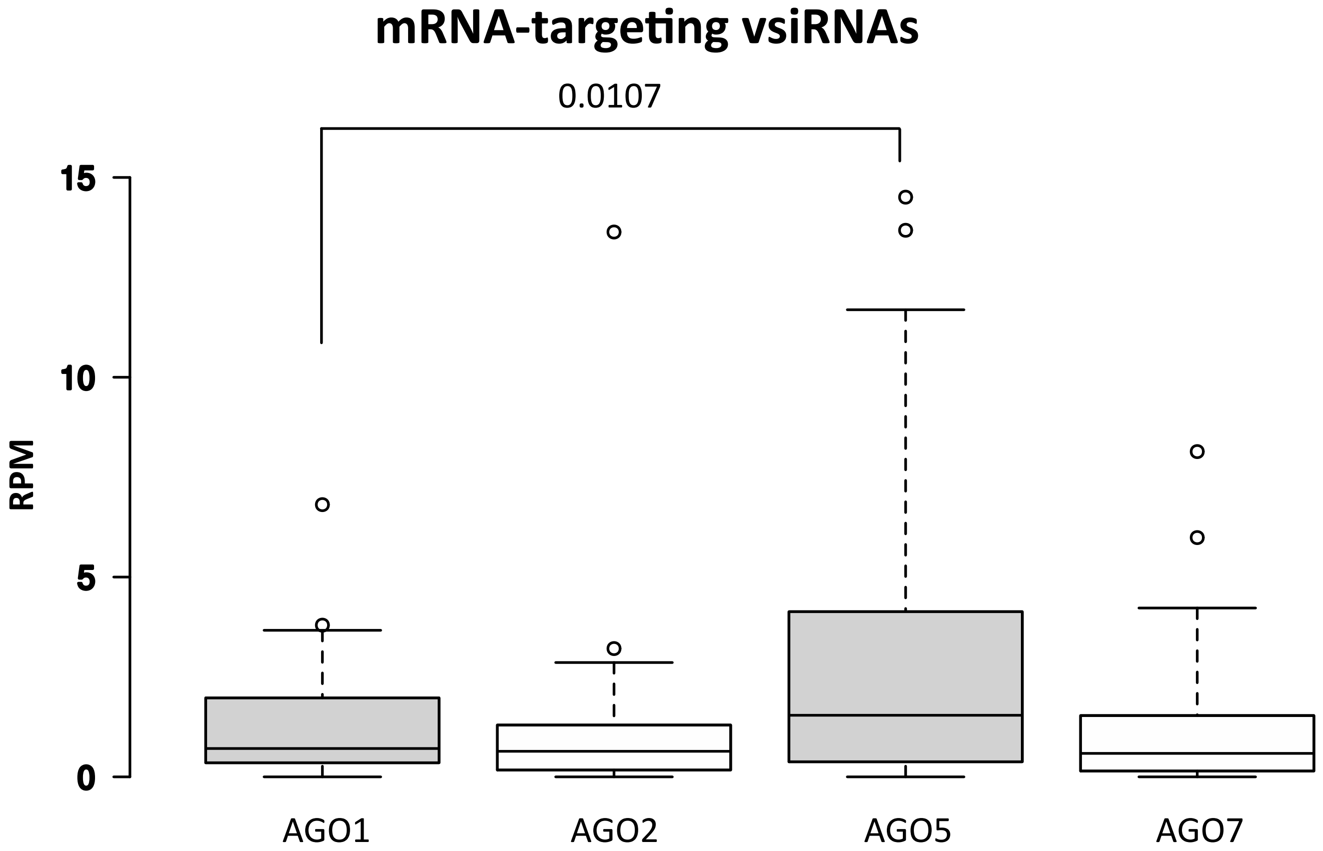
mRNA-targeting vsiRNAs
0.0107
15
10
5
0
RPM
AGO1
AGO2
AGO5
AGO7

Figure S8

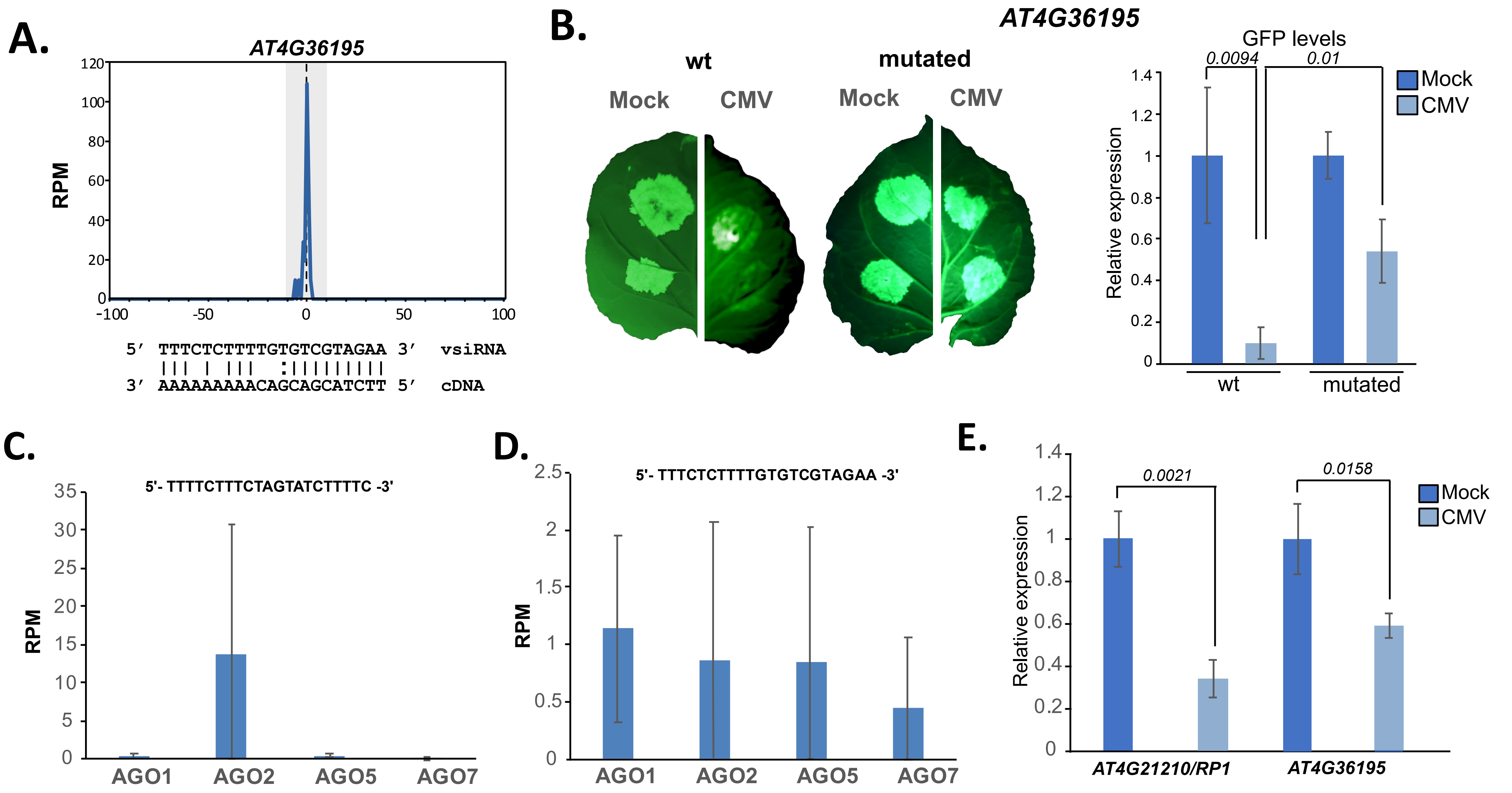

A.
AT4G36195
RPM
120
100
80
60
40
20
0
-100
-50
0
50
100
5′ TTTCTCTTTTGTGTCGTAGAA 3′ vsiRNA
3′ AAAAAAAAACAGCAGCATCTT 5′ cDNA
B.
AT4G36195
wt
mutated
Mock
CMV
Mock
CMV
GFP levels
Relative expression
0.0094
0.01
1.4
1.2
1
0.8
0.6
0.4
0.2
0
Mock
CMV
wt
mutated
C.
5'- TTTTCTTTCTAGTATCTTTTC -3'
RPM
35
30
25
20
15
10
5
0
AGO1
AGO2
AGO5
AGO7
D.
5'- TTTCTCTTTTGTGTCGTAGAA -3'
RPM
2.5
2
1.5
1
0.5
0
AGO1
AGO2
AGO5
AGO7
E.
Relative expression
1.4
1.2
1
0.8
0.6
0.4
0.2
0
0.0021
0.0158
Mock
CMV
AT4G21210/RP1
AT4G36195

Figure S9

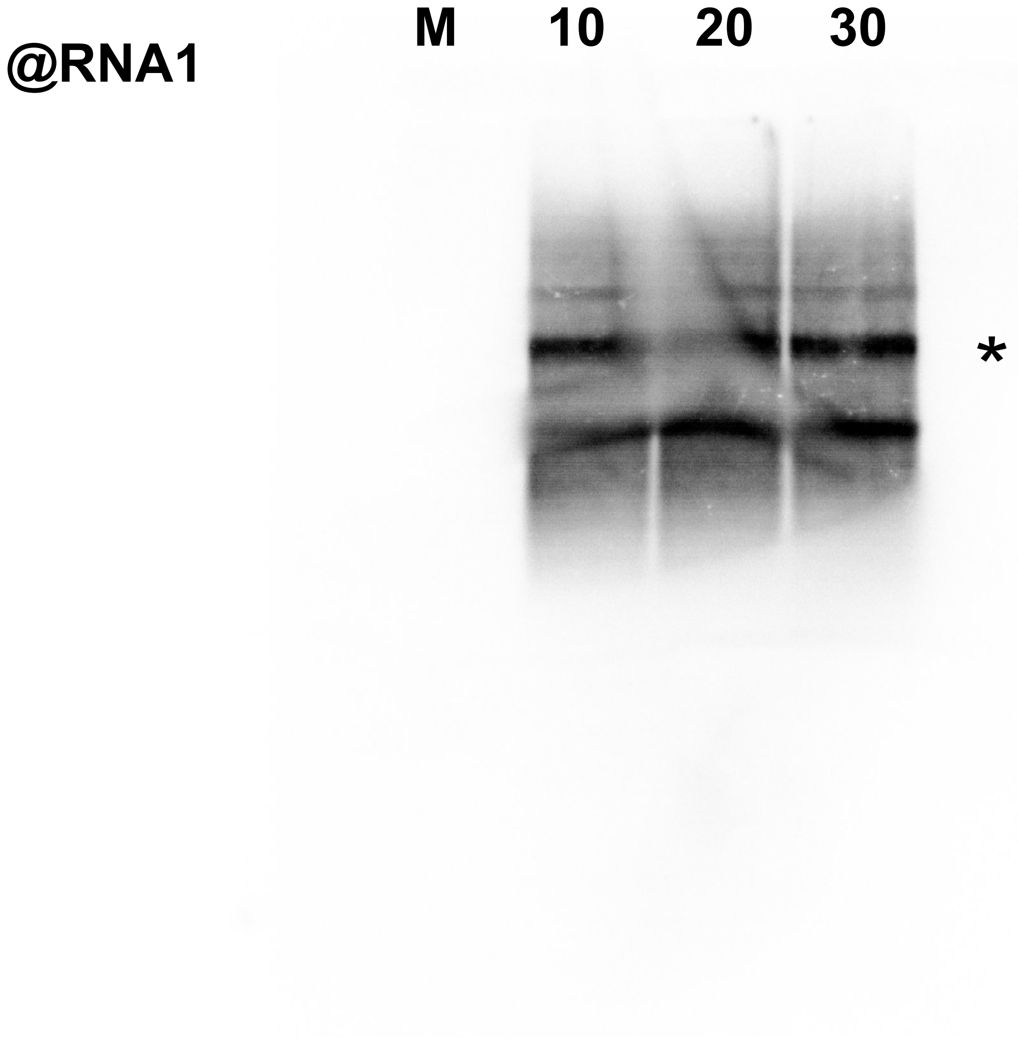

Figure S10

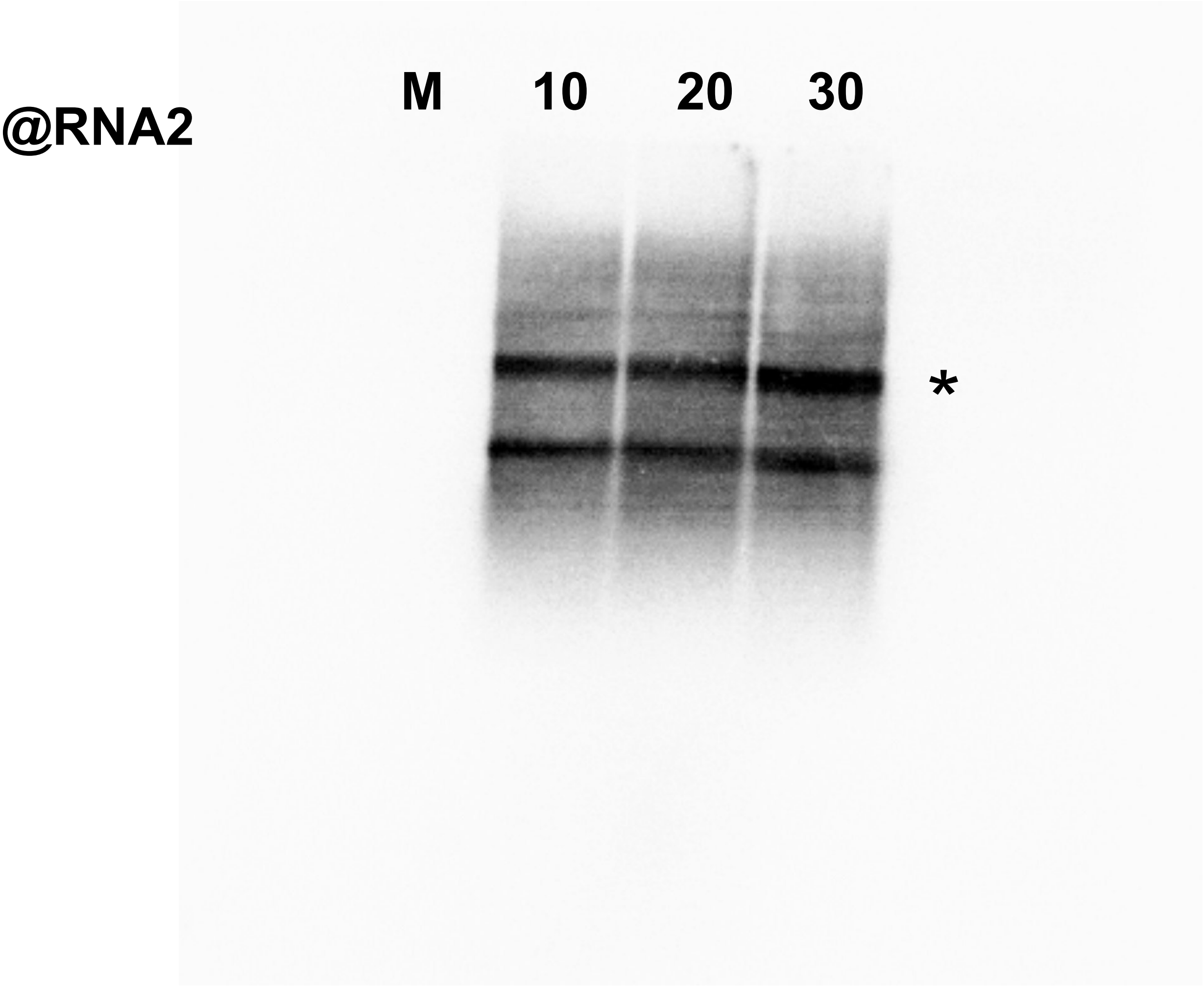

Figure S11

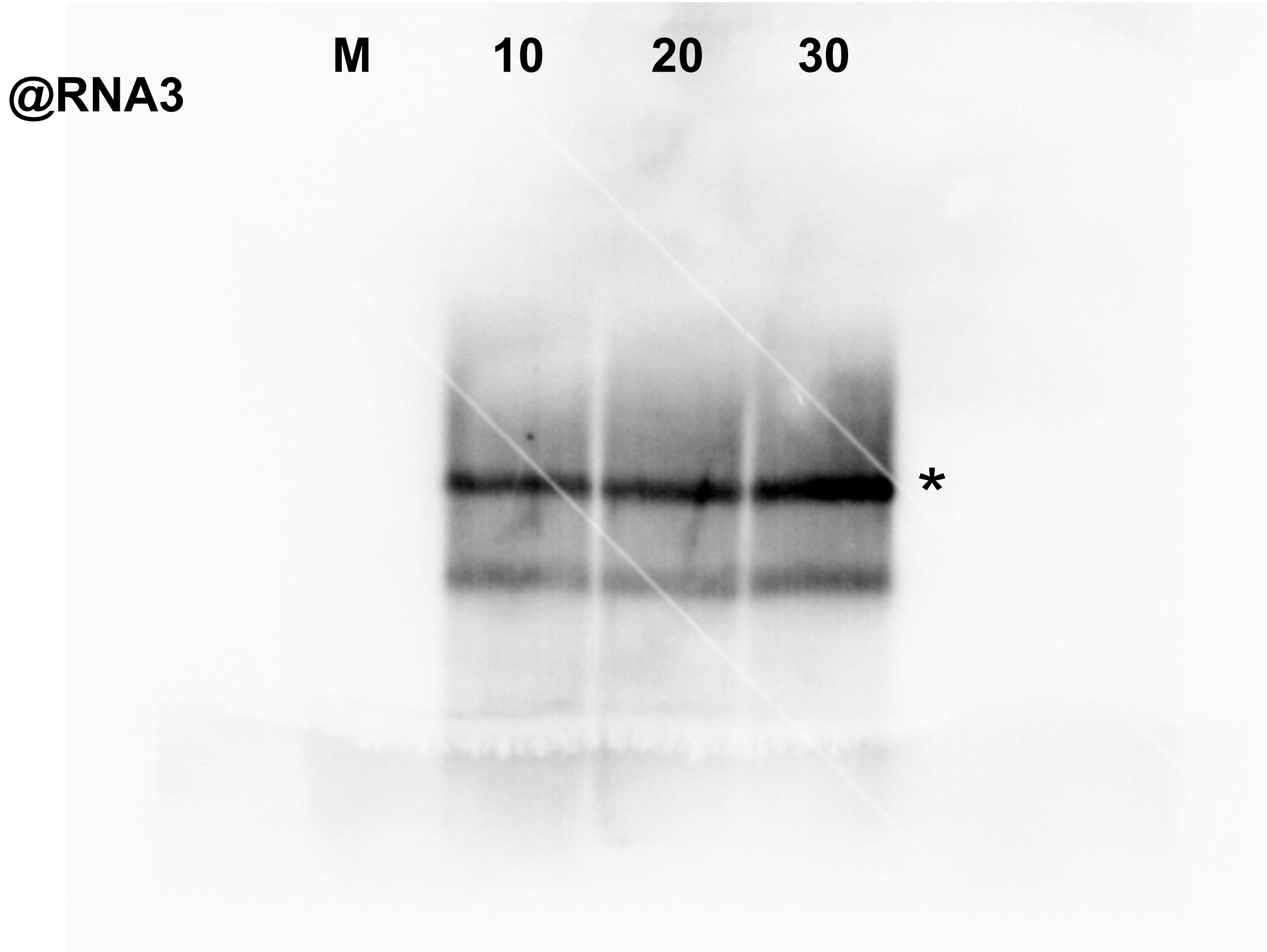

Figure S12

@RNA1

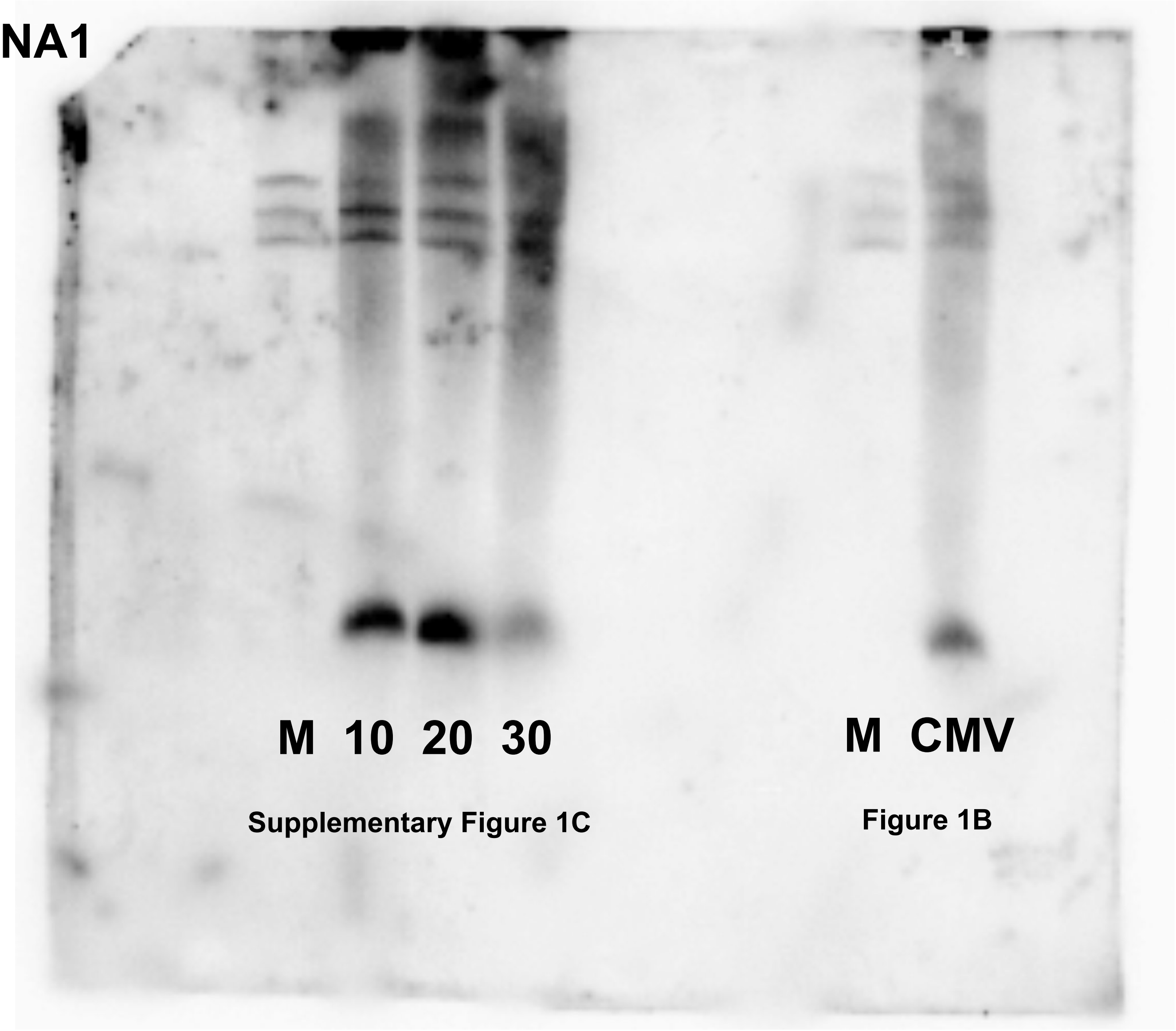

Figure S13

@RNA2

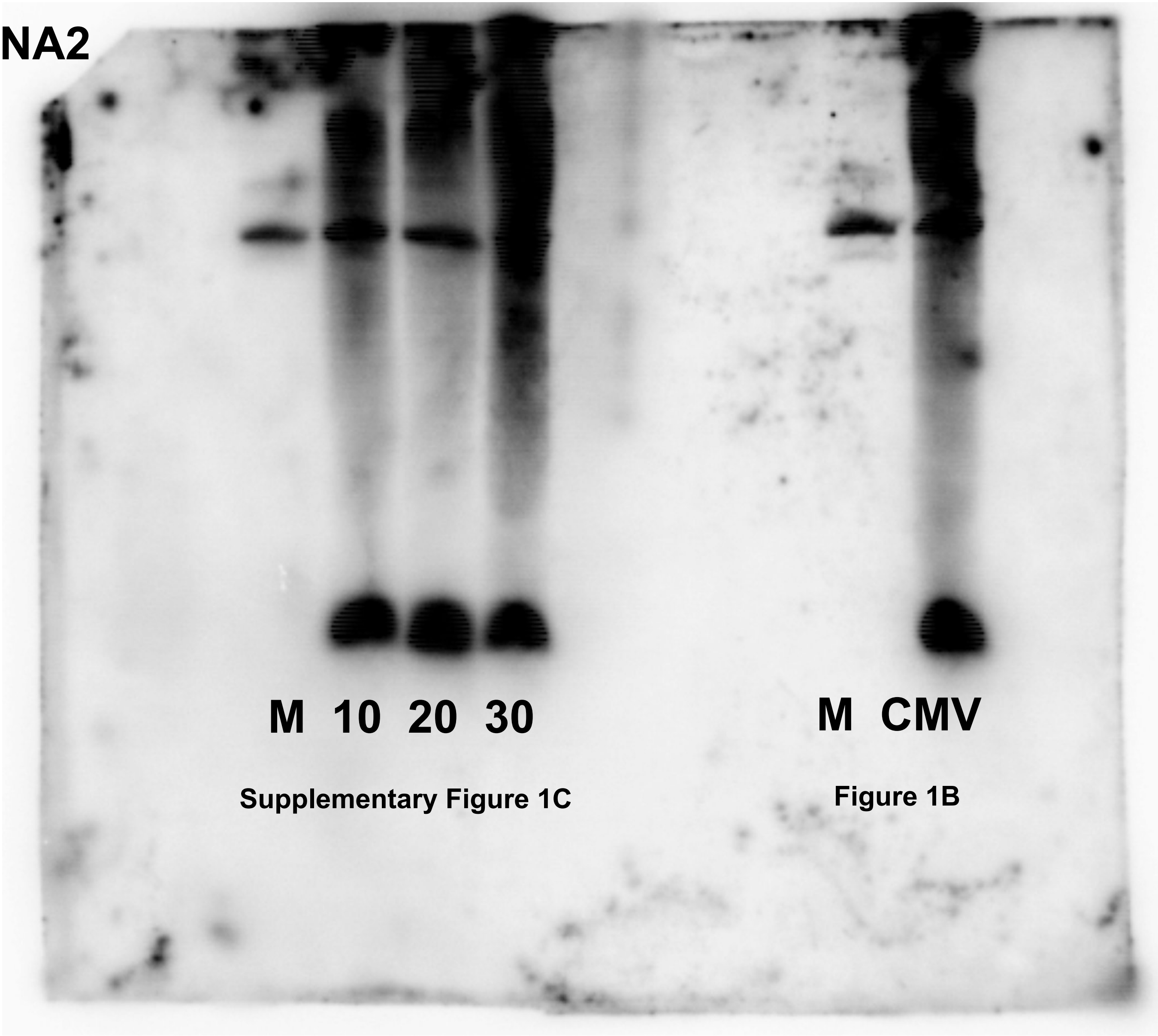

Figure S14

@RNA3

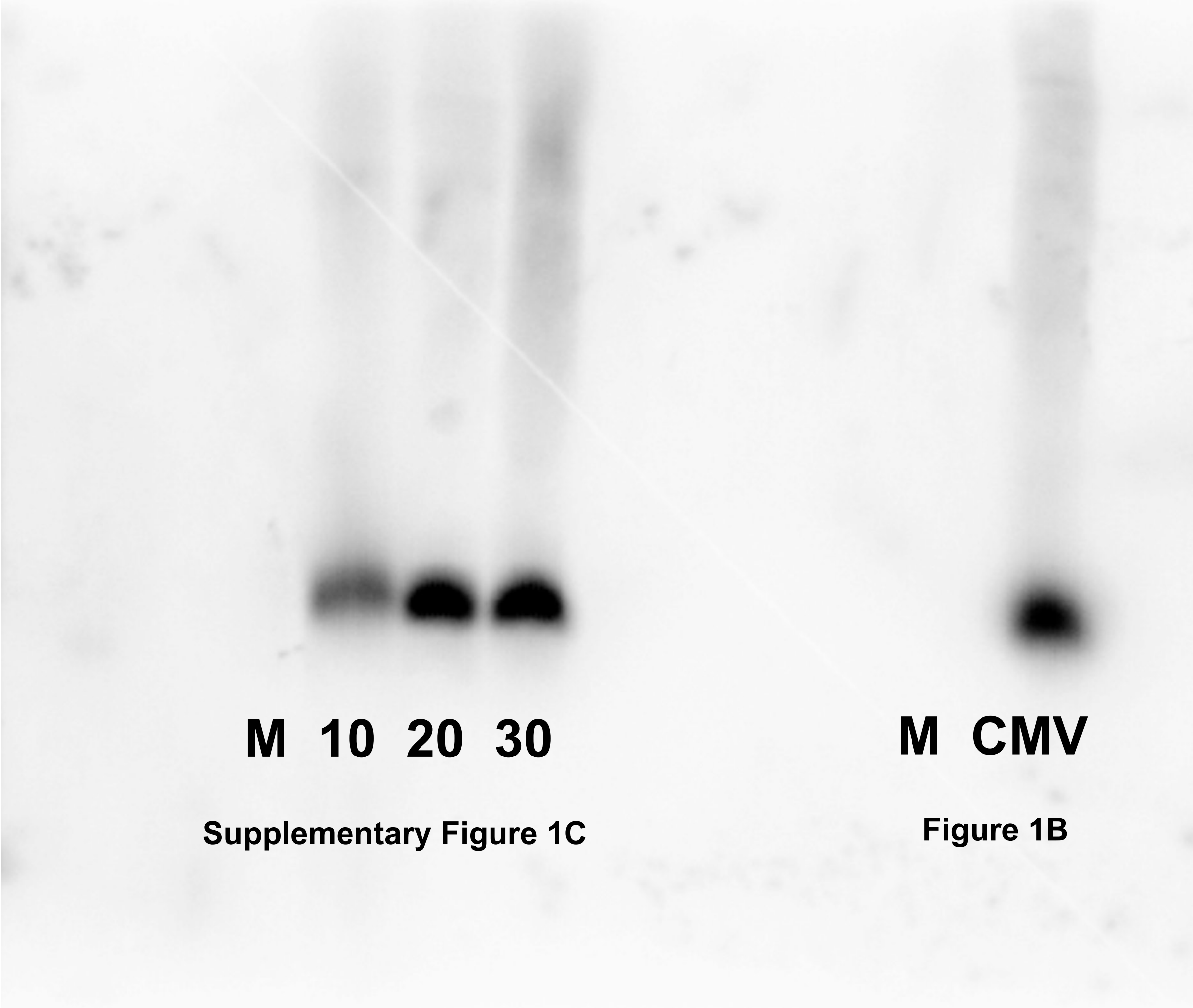

Figure S15

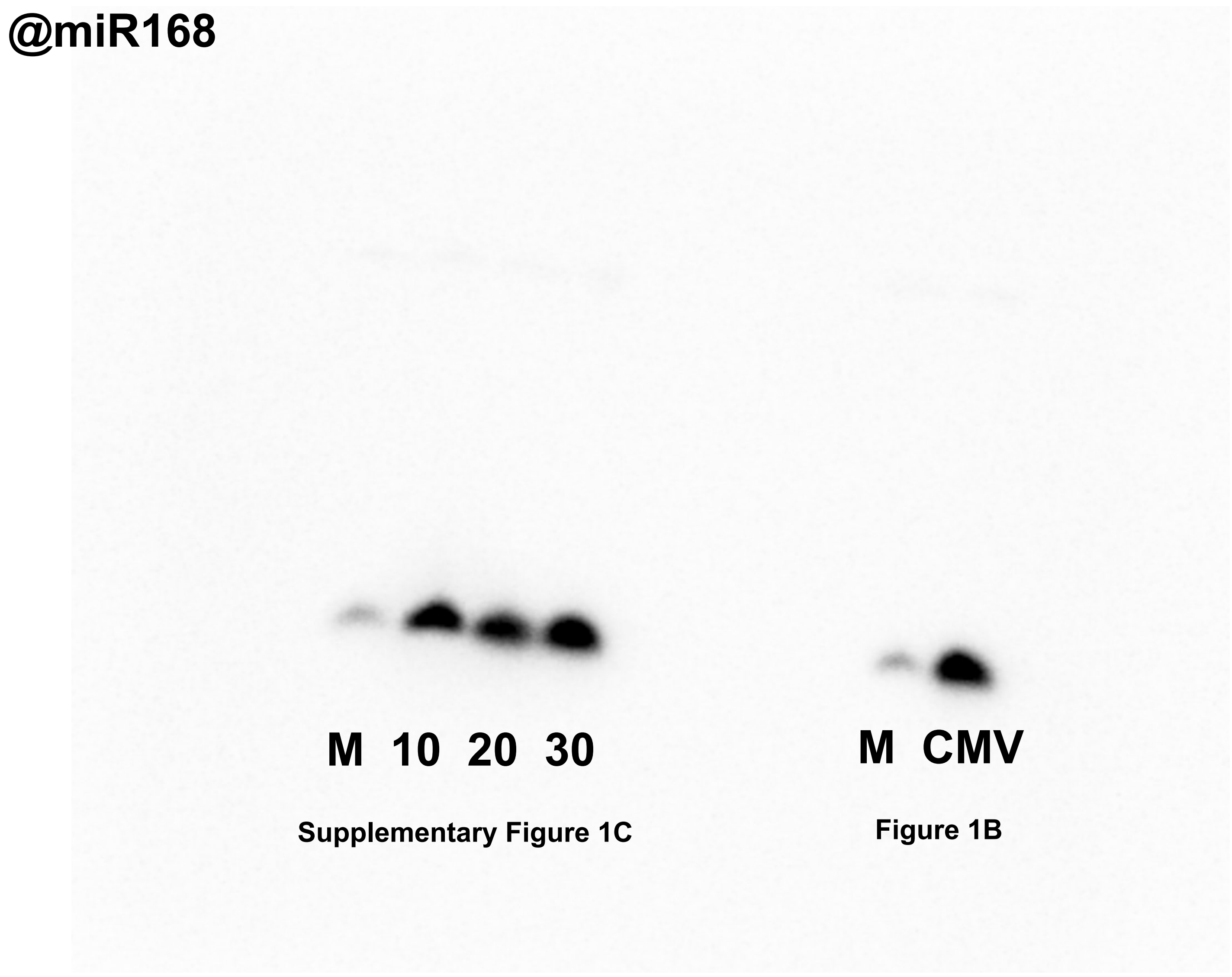

Figure S16

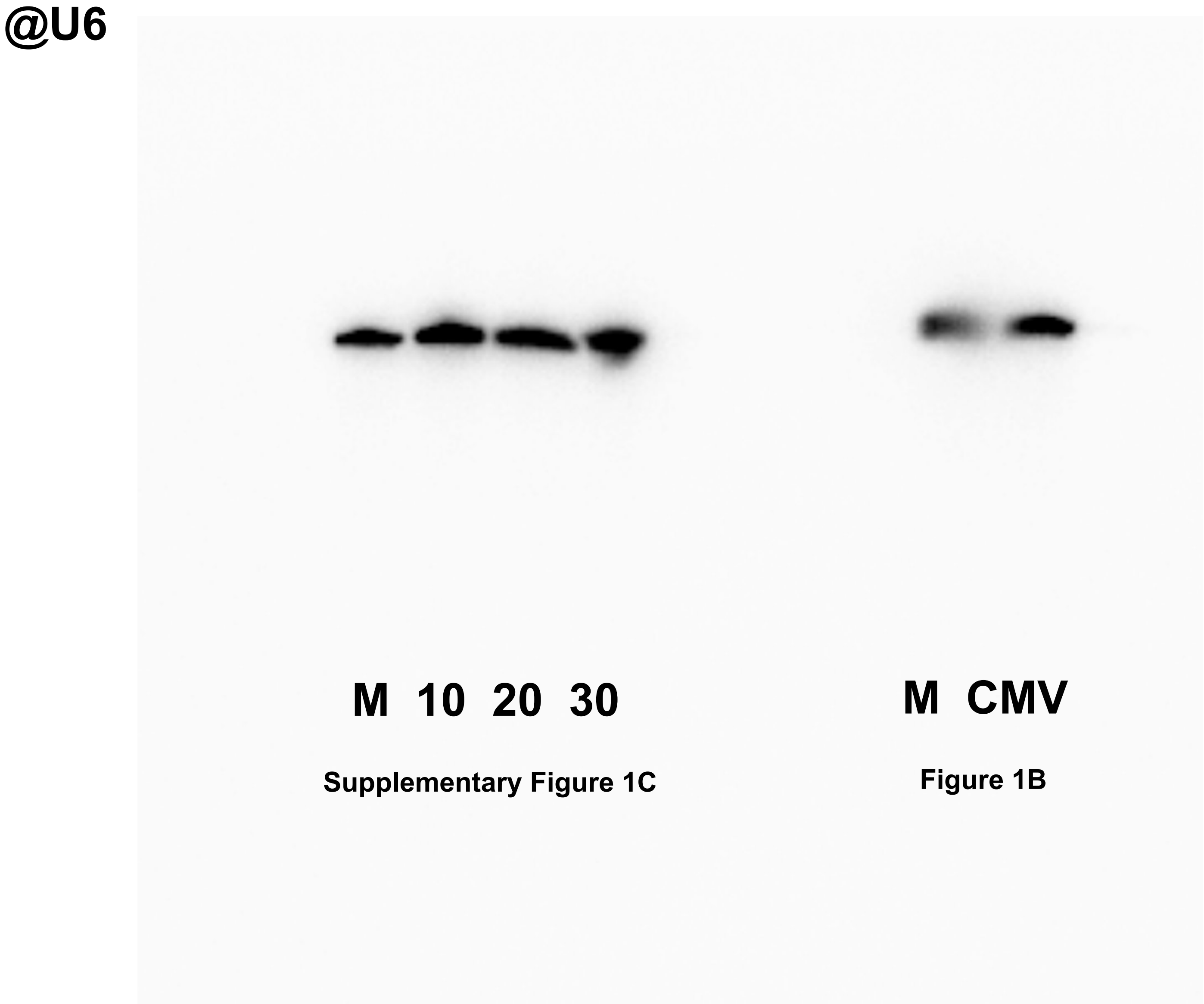

Figure S17

@EtBr

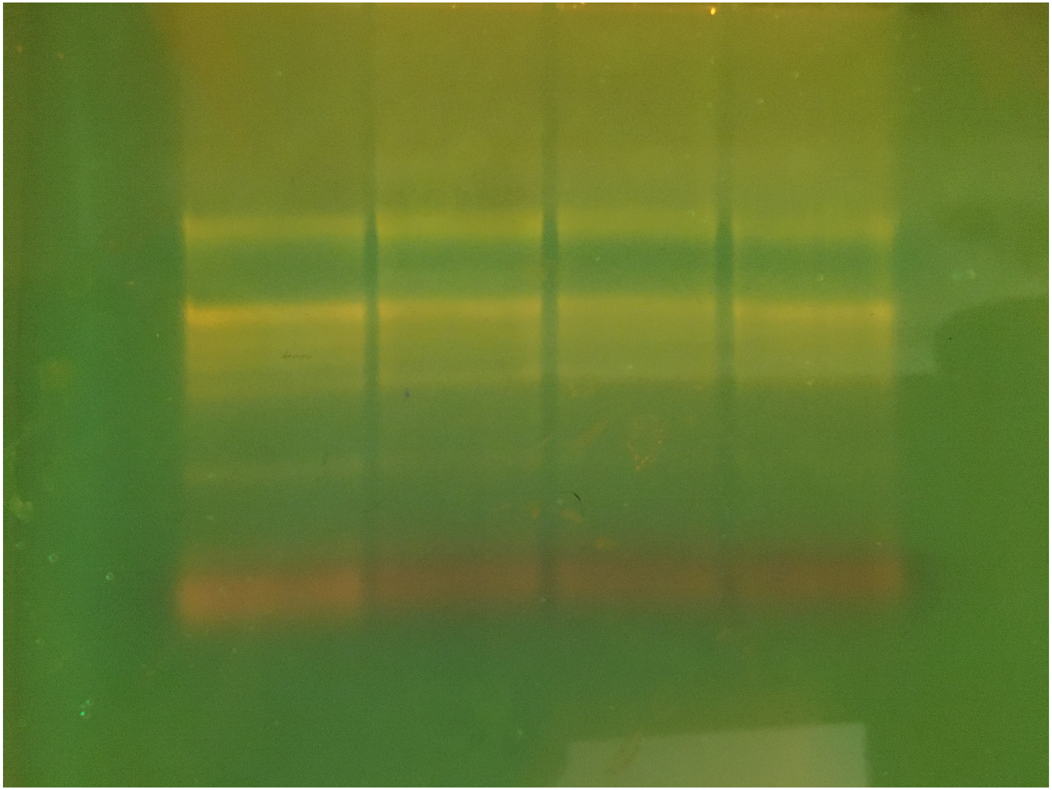

M 10 20 30

Supplementary Figure 1C

Figure S18

@EtBr

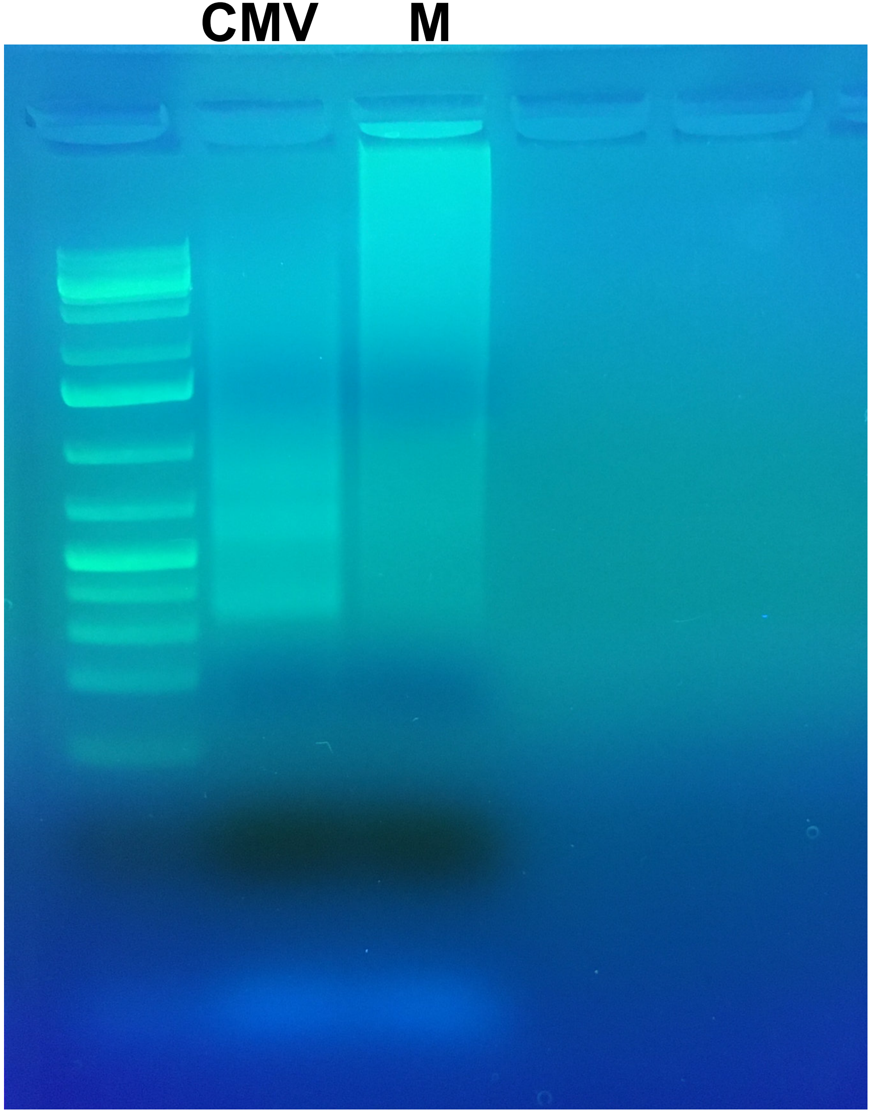

Supplementary Figure 19

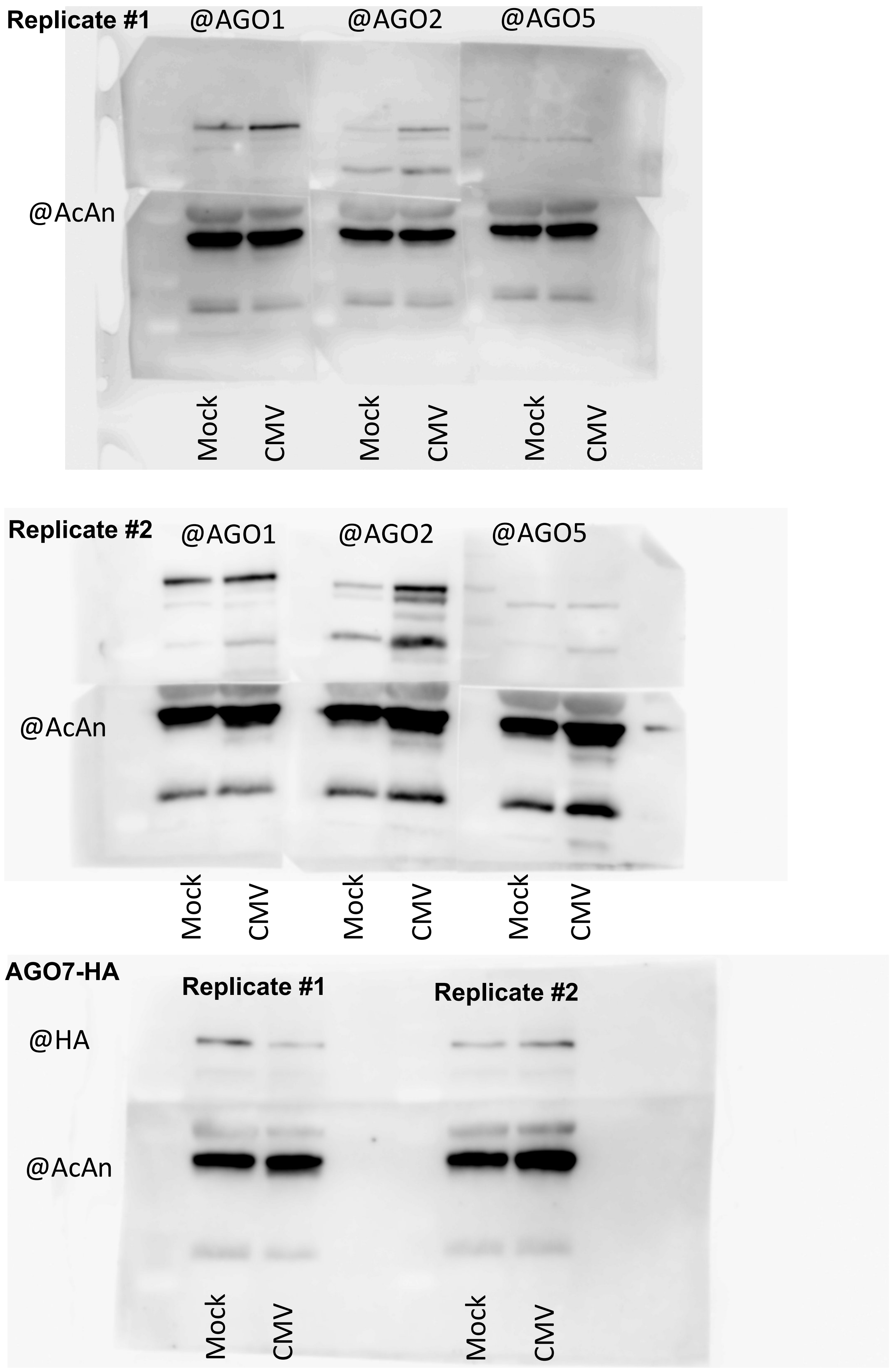

Supplement: Supplementary file 2 — Additional file 2: Supplementary figures. Figure S1. Origin of CMV-derived vsiRNAs. Figure S2. Characterization of endogenous sRNA libraries from mock and CMV-infected tissues. Figure S3. Characterization of TEs producing increased 21-nt sRNAs under CMV infection. Figure S4. AGO antiviral activity and accumulation during CMV infection. Figure S5. Heat map of miRNA accumulation in different AGO-IP sRNA libraries for mock and infected tissues. Figure S6. Characteristics of 2b-IPed sRNAs. Figure S7. Accumulation in different AGOs of vsiRNA targeting mRNAs identified by PARE sequencing. Figure S8. Characteristics of selected vsiRNAs and their targeted genes. Figure S9. Uncropped Northern blot for the detection of CMV genomic RNA1. Figure S10. Uncropped Northern blot for the detection of CMV genomic RNA2. Figure S11. Uncropped Northern blot for the detection of CMV genomic RNA3. Figure S12. Uncropped Northern blot for the detection of vsiRNAs derived from CMV genomic RNA1 Figure S13. Uncropped Northern blot for the detection of vsiRNAs derived from CMV genomic RNA2. Figure S14. Uncropped Northern blot for the detection of vsiRNAs derived from CMV genomic RNA3. Figure S15. Uncropped Northern blot for the detection of miR168. Figure S16. Uncropped Northern blot for the detection of the snRNA U6. Figure S17. Uncropped ethidium bromide-stained agarose gel used as loading control for the detection of CMV genomic RNAs. Figure S18. Uncropped ethidium bromide-stained agarose gel used for the analysis and cloning of 5’RACE fragments derived from AT4G21210. Figure S19. Uncropped Western blot gels used in the detection and quantification of AGO and Actin proteins. [file 13059_2021_2564_MOESM2_ESM.pdf]
