# Supplementary material for: Reprogramming of RNA silencing triggered by cucumber mosaic virus infection in Arabidopsis
Source: Genome Biol. 2021 Dec 15;22:340. doi: 10.1186/s13059-021-02564-z (PMC8672585; doi:10.1186/s13059-021-02564-z)
Supplement: Supplementary file 3 — Additional file 3. Review history. [file 13059_2021_2564_MOESM3_ESM.docx]

Review History

**First round of review**

**Reviewer 1**

**Are you able to assess all statistics in the manuscript, including the appropriateness of statistical tests used?** No, I do not feel adequately qualified to assess the statistics.

**Comments to author:**

In this manuscript, Annacondia and Martinez generate a global overview of the sRNA populations and their incorporation in anti-viral AGO proteins as well as in the VSR 2b during the Cucumber mosaic virus (CMV) infection in Arabidopsis thaliana. The authors also predict that some vsiRNA may target host genes, and confirm this to be the case for a few selected targets. Although some of the aspects featured in this work have been previously described, the approach followed by the authors makes this study unique in that it provides simultaneous information about the effect of the viral infection on sRNA populations, loading, and the potential impact on the plant silencing landscape.
This work in interesting and timely; nevertheless, I have some comments, mostly pertaining the interpretation of the data, that the authors may consider.
Major comments:
- One general question is what is the percentage of Arabidopsis cells infected by CMV in an average infection? Or, in other words, how diluted may these results be, since they will come from a mixture of infected and uninfected cells? This is not something the authors can change at this point, but it might be interesting to at least consider and discuss it.
- Regarding the origin of vsiRNA, does the relative amount correlate with the relative abundance of the corresponding viral RNA? Ideally, this should be experimentally determined and discussed.
- In the infection experiments on ago mutants, how is susceptibility evaluated? A WT plant must be shown alongside, otherwise the results are difficult to interpret.
- While the increased accumulation of AGO2 during the viral infection is clear, an increase of the other AGO proteins is dubious. How many times have the authors performed this experiment, and what are the numbers for each replicate? How is the quantification made (what is used as reference)?
- In Figure S6C, D, there is no difference between WT and ago1, 2.
- Does the down-regulation (or loss of function) of the genes tested in Figure S6 affect performance of the virus? What about lower chlorophyll accumulation?
- Is the TE population with increased derived sRNA enrich in any particular class of TE? Is this increase due to a reactivation of these TEs? Does it depend on 2b?
- In Figure 1B, what is the loading control?
- A large part of the Discussion section is a repetition of the results. Although summarizing the results can help, it would be good to use this section to discuss the potential meaning of the results, as well as the remaining open questions, more in depth.
- L145-147: Is there an image of this northern blot?

Minor comments:
- Language must be revised - there are small mistakes/typos/omissions scattered throughout the text. Some examples: L53 - "their" meaning…; L56/57: repetition of "mechanism"; L74 - "important"?; L109-110 - repetition of "described"; L143 - "led"?; L173 and elsewhere - "especially"?; L238 - "present"; L336 - "of"; L352 - "are"?; L382 - "led"?
- L86-88: Wouldn't the interaction with AGO4 be expected to affect TGS, not PTGS?
- What to the authors mean in L170 with "According to this antiviral role"?
- In L213-214, could the authors be more specific when they mention "developmental defects compatible with the inhibition of RNA silencing pathway"?
- In L237, I don't understand what the authors mean.
- In Figure 1D, the colours for CMV-infected plants could be more different. Is the order swapped in legend and bars?
- In Figure 1E, could the authors indicate %?
- A reference to Ando et al., 2021, MPP, may be perhaps useful.
- L82-83: Isn't this pathway triggered by dsRNA and not by the siRNAs, which are products deriving from the processing of this dsRNA?
- L133-136: And 24-nt sRNA? The authors exclude this species form analysis or discussion, even though 2b has been shown to interact with AGO4 (Gonzalez et al., 2010); the reason for this should be clearly stated.
- In Figure 2D and others, in the figure legend for the different RNA species, the ones derived from CMV should be indicated as "vsiRNA" or "CMV siRNAs", but not as "CMV".
- L207 - Could this be a "displacement" rather than "preferential loading"?
- In Figure 4B, the numbers in the x axis are displaced to the left and do not fit their proper location, please move to the right.
- L271 - Actually, for this elongation factor Tu (AT5G60390), the column on the right (in Figure 4A) indicates that the expression level is increased during the CMV infection, not decreased?
- L288-290 - From these two genes, AT4G21210 (RP1) and AT4G36195, only RP1 exhibits lower expression levels in CMV infected plants (see Figure 4A, column on the right), while AT4G36195 increases or remains unchanged. So, the behavior the authors show for the gene AT4G36195 in Figure 5E vs Figure 4A is the opposite... Is there some mistake in Figure 4A?
- Figure S6A and B: since the graphs show RPM counts, where do the error bars come from? Please explain this in the figure legend.
- The statistical analyses shown in Figure 5C and 5D and Figure S6C and 6D do not support the interpretation given to these results.
- Please indicate the number of replicates used for all experiments in the corresponding figure legends.
- Please double-check the vsiRNA-targeting sequences in the different figures and tables and make sure they are consistent.

**Reviewer 2**

**Are you able to assess all statistics in the manuscript, including the appropriateness of statistical tests used?** No, I do not feel adequately qualified to assess the statistics.

**Comments to author:**

The manuscript submitted by Maria Luz Annacondia et al. entitled "Reprograming of RNA silencing triggered by Cucumber mosaic virus infection in Arabidopsis" reported their study on CMV derived small RNAs (vsiRNA), in which they found that production of vsiRNAs affect other sRNA classes, and the small RNAs that loaded into AGO proteins including AGO1, AGO2, AGO5, and AGO7, and small RNAs bound by 2b protein were then sequenced. Very interestingly, they found that vsiRNAs represented a significant percentage of AGO-loaded vsiRNAs. Furthermore, they have analyzed the endogenous potential targets of vsiRNAs, which loaded into the RISC complex may play important roles in antiviral defense. The experiments is well designed and the results are interesting and well presented. The paper is well written. But before acceptance for publication, there are some issues needed to be addressed.
Major:
Although the authors applied several methods to confirm the endogenous potential targets of vsiRNAs, the RACE results are still required to validate the biological functions of vsiRNAs in the host under viral infection condition.

Minor:
1. In Fig. 1B, a loading control is needed.
2. Refence 65 and 35 are the same.
3. Refence 64 and 12 are the same.

**Authors Response**

**Reviewers’ report answered:**

**Reviewer #1**: In this manuscript, Annacondia and Martinez generate a global overview of the sRNA populations and their incorporation in anti-viral AGO proteins as well as in the VSR 2b during the Cucumber mosaic virus (CMV) infection in Arabidopsis thaliana. The authors also predict that some vsiRNA may target host genes, and confirm this to be the case for a few selected targets. Although some of the aspects featured in this work have been previously described, the approach followed by the authors makes this study unique in that it provides simultaneous information about the effect of the viral infection on sRNA populations, loading, and the potential impact on the plant silencing landscape.

This work in interesting and timely; nevertheless, I have some comments, mostly pertaining the interpretation of the data, that the authors may consider.

**Major comments:**

- One general question is what is the percentage of Arabidopsis cells infected by CMV in an average infection? Or, in other words, how diluted may these results be, since they will come from a mixture of infected and uninfected cells? This is not something the authors can change at this point, but it might be interesting to at least consider and discuss it.

- Thanks a lot for bringing up this aspect, which is very interesting. The parameter that the reviewer is referring to has been termed cellular multiplicity of infection (MOI). Although MOI is unknown for CMV it is known for other monopartite RNA viruses such as *Tobacco etch virus* (TEV), *Tobacco mosaic virus* (TMV) or *Turnip mosaic virus* (TuMV). Monopartite viruses are easier to study in this regard since they can be fused to reporter genes, which helps to study the number of cells studied, as well as the number of genomes per cell. A summary of the reports (which are very limited) about accumulation of viral genomes on infected tissues, it seems that between 1 or 2 viral genomes accumulate in an infected cell at later infection times, while those numbers can be as high as 6 at earlier infection points. The number of cells infected by a virus has been less studied, with numbers varying from 7 to 42% of all cells infected by a virus. Our samples come from relatively later times points (30 dpis), but we see a stable accumulation of the viral genome (confirmed by Northern blot analysis) which is not correlated with vsiRNA accumulation, which is more dynamic during infection. We think that this lack of correlation shows perfectly the spread of the antiviral RNA silencing signal and RDR6-mediated amplification of vsiRNAs. We added these concepts to the discussion of the paper. Northern blots can be seen in Supplementary Figure 1C.

- Regarding the origin of vsiRNA, does the relative amount correlate with the relative abundance of the corresponding viral RNA? Ideally, this should be experimentally determined and discussed.

-We have now analyzed by Northern blot CMV genomic RNAs and vsiRNA accumulation at three different infection times: 10, 20 and 30 dpi. As commented above, we observed a stable accumulation of the viral genome (by Northern blot) which is not correlated with vsiRNA accumulation, which seems to be dynamic during infection. We argue that this lack of correlation indicates the spread of the antiviral vsiRNAs and their amplification by RDRs. This data is also in line with previous analysis of RDR influence over CMV-vsiRNA accumulation from Professor Shou-wei Ding’s group (Wang et al (2010) PNAS). We added these concepts to the discussion of the paper. Northern blots can be seen in Supplementary Figure 1C.

- In the infection experiments on ago mutants, how is susceptibility evaluated? A WT plant must be shown alongside, otherwise the results are difficult to interpret.

-We have now performed a quantification of the symptomatology in AGO mutants for all the AGOs analyzed in our work. We quantified how the viral infection affects the rosette radius of infecting plants by quantifying the ratio CMV/Mock, which is a known parameter affected by CMV infection. Our results show that AGO1, AGO2 and AGO5 are hypersusceptible to the infection, while AGO7 is slightly tolerant to the infection. This new data is presented in a new supplementary figure (Supplementary Figure 3A).

- While the increased accumulation of AGO2 during the viral infection is clear, an increase of the other AGO proteins is dubious. How many times have the authors performed this experiment, and what are the numbers for each replicate? How is the quantification made (what is used as reference)?

-We have repeated the Western blot detection of AGO proteins during infection twice. This data is now shown in Supplementary Figure 3B.

- In Figure S6C, D, there is no difference between WT and ago1, 2.

-We are aware of this. We think that these lack of return to WT values of expression is due to the known redundancy between AGO proteins. To avoid any misunderstood, we have now removed that panel.

- Does the down-regulation (or loss of function) of the genes tested in Figure S6 affect performance of the virus? What about lower chlorophyll accumulation?

-We have tried to obtain homozygous mutants for both genes but it has been impossible for us to identify any mutant in the alleles that we have requested from NASC (2 for RP1 and 1 for AT4G36195). The current COVID and Brexit situations delayed the delivery of seeds and we also identify problems with the germination of some of these lines. We are currently working to identify how important these genes are for viral infection but our results are too preliminary. RP1 is the best described of both genes and its role in chlorophyll accumulation is well described in Arabidopsis (check references Chastain et al (2007) The Plant Journal and Astley et al (2011) The Plant Journal). We have added references to this works in the main text and the discussion, where we added a paragraph regarding the common targeting of photosynthesis-associated genes by viral and subviral pathogens and speculating about the potential role of this.

- Is the TE population with increased derived sRNA enrich in any particular class of TE? Is this increase due to a reactivation of these TEs? Does it depend on 2b?

-We thank the reviewer for this question, which opened a very interesting perspective to our data. First, we have used public datasets to check the epigenetic status of TEs that produce 21-nt sRNAs under CMV infection. Additionally, to check the influence of 2b in the production of these sRNAs, we have analyzed the production of 21-nt TE-derived sRNAs in data from Professor Shou-wei Ding’s lab, which was obtained using a CMV strain mutant for the 2b protein (known as CMV-∆ 2b). In summary, our analysis of this data shows that 21-nt sRNA production from these TEs depends on the presence of 2b in the CMV genome, and that these TEs are very heterochromatic, since they have high values of both DNA methylation and H3K9me2. According to their heterochromatic nature, they are longer in average than the overall population of TEs in *Arabidopsis* and are enriched Gypsy TEs, known to be located in pericentromeric and centromeric regions. This new analysis is shown in a dedicated supplementary figure, Supplementary Figure 3.

- In Figure 1B, what is the loading control?

-We have now repeated the Northern blots in Figure 1B and included a proper loading control for sRNAs, the U6 snRNA.

- A large part of the Discussion section is a repetition of the results. Although summarizing the results can help, it would be good to use this section to discuss the potential meaning of the results, as well as the remaining open questions, more in depth.

-In this new version of the manuscript we have completely rewritten the discussion section. We have now included further speculation about the meaning of the results and future directions and open questions.

- L145-147: Is there an image of this northern blot?

-Yes, it is the Northern blot in Figure 1B. We have now fixed the reference to the figure in the main text.

**Minor comments:**

- Language must be revised - there are small mistakes/typos/omissions scattered throughout the text. Some examples: L53 - "their" meaning…; L56/57: repetition of "mechanism"; L74 - "important"?; L109-110 - repetition of "described"; L143 - "led"?; L173 and elsewhere - "especially"?; L238 - "present";  L336 - "of"; L352 - "are"?; L382 - "led"?

-Thanks for highlighting these typos. We have now corrected these typos and tried, to the best of our possibilities, to improve the quality of the text.

- L86-88: Wouldn't the interaction with AGO4 be expected to affect TGS, not PTGS?

-Thanks for highlighting this conceptual mistake. We have now corrected this.

-What to the authors mean in L170 with "According to this antiviral role"?

-Sorry for this mistake. We have now corrected the sentence.

- In L213-214, could the authors be more specific when they mention "developmental defects compatible with the inhibition of RNA silencing pathway"?

- We have now corrected the sentence. Thanks a lot for highlighting this grammatical problem.

- In L237, I don't understand what the authors mean.

- We have now modified the sentence. We meant that 2b makes a significant contribution of vsiRNAs to the overall amount of vsiRNAs accumulating in the cell during infection.

- In Figure 1D, the colours for CMV-infected plants could be more different. Is the order swapped in legend and bars?

-Great suggestion. We have now corrected these colors and the order in the legend.

- In Figure 1E, could the authors indicate %?

-Sorry for the mistake. We have now added a legend to the axis.

- A reference to Ando et al., 2021, MPP, may be perhaps useful.

-Thanks for the tip. We have now added a reference and commentary to this work in the discussion, associated to the potential role of epigenetic changes in the activation of AGO activity during viral infection.

- L82-83: Isn't this pathway triggered by dsRNA and not by the siRNAs, which are products deriving from the processing of this dsRNA?

- We agree with the reviewer in this aspect. We have removed that second part of the original sentence which referred to the results of a manuscript that was retracted.

- L133-136: And 24-nt sRNA? The authors exclude this species form analysis or discussion, even though 2b has been shown to interact with AGO4 (Gonzalez et al., 2010); the reason for this should be clearly stated.

- We added an explanatory paragraph about the changes observed in the accumulation of 24 nt endogenous sRNAs both in the main text and in the discussion. In this manuscript we focused only in the analysis of vsiRNAs and their effects. We are currently working in understanding the connection between the observed changes in 24-nt endogenous sRNAs and global DNA methylation during CMV infection. We thought that our data was too preliminary and out of the focus of this work to show in this manuscript.

- In Figure 2D and others, in the figure legend for the different RNA species, the ones derived from CMV should be indicated as "vsiRNA" or "CMV siRNAs", but not as "CMV".

- Thanks. We have carefully checked all the figures and homogenize terms and colors for the different histograms that show similar categories.

- L207 - Could this be a "displacement" rather than "preferential loading"?

- Certainly. We have borrowed the term from the reviewer comment and incorporated it in the main text.

- In Figure 4B, the numbers in the x axis are displaced to the left and do not fit their proper location, please move to the right.

- We have corrected this problem that was due to the preparation of the figure.

- L271 - Actually, for this elongation factor Tu (AT5G60390), the column on the right (in Figure 4A) indicates that the expression level is increased during the CMV infection, not decreased?

-We have decided to remove the gene from the list of examples mentioned due to this lack of correlation with RNA seq data. The actual fold change for AT5G60390 is 1.2-fold, so it cannot be considered clearly downregulated. Nevertheless, we must highlight that the target genes were selected according to our identification in PARE/degradome sequencing data, which might be slightly different from the RNA sequencing which was obtained from public databases.

- L288-290 - From these two genes, AT4G21210 (RP1) and AT4G36195, only RP1 exhibits lower expression levels in CMV infected plants (see Figure 4A, column on the right), while AT4G36195 increases or remains unchanged. So, the behavior the authors show for the gene AT4G36195 in Figure 5E vs Figure 4A is the opposite... Is there some mistake in Figure 4A?

-In Figure 4A, actually AT4G36195 shows no change in expression during infection (1.3-fold change). Nevertheless, the gene has a clear cleavage peak in the PARE/degradome sequencing corresponding to a cleavage event mediated by a vsiRNA (Supplementary Figure D). We tested expression of both targeted genes by RT-qPCR (Supplementary Figure B) and in both cases our data shows a significant decrease in expression during CMV Fny infection. The differences might be due to the different nature of the samples, since the RNA sequencing data comes from public databases. We have clarified this issue in the main text.

- Figure S6A and B: since the graphs show RPM counts, where do the error bars come from? Please explain this in the figure legend.

-We have 2 bioreplicates of each of the sequencing experiments performed in the manuscript: sRNA, PARE and AGO-sRNA RIP sequencing.

- The statistical analyses shown in Figure 5C and 5D and Figure S6C and 6D do not support the interpretation given to these results.

-We have now moderated our interpretation of the results for both constructs. We introduced 4 mutations in the 3’ of our target site which correspond to the 5’ seed of the vsiRNA-mRNA target interaction. It is plausible that despite introducing these mutations the vsiRNA is still able to bind to the target site and induce some cleavage of the gene. Indeed, some differences in RNA accumulation is still observed between the introduction of 4, 5 and 6 mismatches in the thorough analysis of miRNA cleavage performed by Liu et al (2014) The Plant Cell. We added a comment regarding this in the manuscript. Additionally, we have included a 5’RACE analysis of the targeting of RP1 by its corresponding vsiRNA, which confirms the cleavage position identified by PARE. This new analysis is shown in Figure 5B.

- Please indicate the number of replicates used for all experiments in the corresponding figure legends.

-We have now indicated the number of bioreplicates for each experiment analyzed in the figure legends. Number of bioreplicates is also indicated in Supplementary Figure 1.

- Please double-check the vsiRNA-targeting sequences in the different figures and tables and make sure they are consistent.

-We have now made sure that all vsiRNA sequences are in 5’->3’ orientation

**Reviewer #2**: The manuscript submitted by Maria Luz Annacondia et al. entitled "Reprograming of RNA silencing triggered by Cucumber mosaic virus infection in Arabidopsis" reported their study on CMV derived small RNAs (vsiRNA), in which they found that production of vsiRNAs affect other sRNA classes, and the small RNAs that loaded into AGO proteins including AGO1, AGO2, AGO5, and AGO7, and small RNAs bound by 2b protein were then sequenced. Very interestingly, they found that vsiRNAs represented a significant percentage of AGO-loaded vsiRNAs. Furthermore, they have analyzed the endogenous potential targets of vsiRNAs, which loaded into the RISC complex may play important roles in antiviral defense. The experiments are well designed, and the results are interesting and well presented. The paper is well written. But before acceptance for publication, there are some issues needed to be addressed.

**Major:**

Although the authors applied several methods to confirm the endogenous potential targets of vsiRNAs, the RACE results are still required to validate the biological functions of vsiRNAs in the host under viral infection condition.

-We have now followed the suggestion of the reviewer and we have confirmed one of our vsiRNA-targeted genes by modified 5’ RACE. This data is now shown in Figure 5B and includes a control of the 5’RACE amplification (lack of amplification in this case) in mock tissue. Unfortunately, we couldn’t confirm the other targeted gene since we only found 5’RACE fragments that were downstream of the vsiRNA-targeted position. It might be possible that the fragments derived from this targeting event accumulate to very low levels and are only detected at significant level using high-throughput sequencing. For this reason, we have decided to move the data of the targeting of AT4G36195 to Supplementary Figure 8.

Minor:

1. In Fig. 1B, a loading control is needed.

-We agree with the reviewer. In our original version we used miR156 which did not change its accumulation during CMV infection. In this new version of the manuscript we have used the more reliable U6 snoRNA as a loading control of RNA.

2. Reference 65 and 35 are the same.

3. Reference 64 and 12 are the same.

- We thank the reviewer for spotting these duplicated references. We have double-checked the bibliography to avoid these mistakes.

**Second round of review**

**Reviewer 1**

I would like to congratulate the authors for the commendable effort they have made to improve the manuscript and incorporate all of the reviewers´ comments, despite the numerous challenges of the current times - both the response to reviewers and the manuscript itself have been a pleasure to read. (I would also like to thank them, because this revised version has just made my day!).
